# Total synthesis and antimicrobial evaluation of (+)-hygrophorone B^12^ and its analogues

**DOI:** 10.1038/s41598-022-11608-8

**Published:** 2022-05-06

**Authors:** Takaaki Kamishima, Masato Suzuki, Koichi Narita, Yoshitaka Koseki, Toshiyuki Nonaka, Hirotaka Nakatsuji, Hideo Hattori, Hitoshi Kasai

**Affiliations:** 1 East Tokyo Laboratory, Genesis Research Institute, Inc., 717-86 Futamata, Ichikawa, Chiba 272-0001 Japan; 2 Antimicrobial Resistance Research Center, National Institute of Infectious Diseases, 4-2-1 Aobamachi, Higashimurayama, Tokyo, 189-0002 Japan; 3 Faculty of Pharmaceutical Sciences, Tohoku Medical and Pharmaceutical University, 4-4-1 Komatsushima, Aoba-ku, Sendai, Miyagi 981-8558 Japan; 4 Institute of Multidisciplinary Research for Advanced Materials, Tohoku University, 2-1-1 Katahira, Aoba-ku, Sendai, Miyagi 980-8577 Japan; 5 Fromseeds Corporation, 6-6-40 Aramaki, Aoba-ku, Sendai, Miyagi 980-0845 Japan

**Keywords:** Natural product synthesis, Drug development, Structure-based drug design

## Abstract

This paper describes the synthesis and evaluation of lead compounds with a new chemical skeleton that is not found in conventional antimicrobial agents. The biologically attractive cyclopentenoid (+)-hygrophorone B^12^, isolated from the fruiting bodies of *Hygrophorus abieticola*, and its analogues were synthesized in a longer linear sequence of twelve steps, starting from a cyclopentenone derivative. This synthesis involved the following crucial steps: (i) oximation of a ketone to stabilize the requisite aldehyde to install a side chain and (ii) coupling of an aldehyde with a side chain to assemble the desired hygrophorone. Then, the antimicrobial activity of these hygrophorones towards clinically relevant bacterial pathogens was evaluated. The results showed that hygrophorone B^12^ and its analogues are especially effective in preventing the proliferation of gram-positive bacteria. In addition, it was found that some structural features such as the presence of the enone moiety as well as the carbon–carbon triple bond on the hydrocarbon chain were pivotal to increase the antimicrobial activity of hygrophorone B. This study is expected to support the development of novel antimicrobial agents by flexibly synthesizing hygrophorone B analogues with a carbon five-membered ring skeleton from the common intermediate.

## Introduction

Various antibiotics and other antimicrobial agents have been developed and they have saved uncountable numbers of lives. However, the threat of antimicrobial resistance (AMR) has recently arisen and must be addressed urgently^1,2^. Even though the problem of morbidity and mortality associated with AMR is not new, AMR has recently been increasing at a significant rate due to bacteria that have acquired resistance to multiple groups of antimicrobial agents^2^. Moreover, the decline in the development and marketing of new antimicrobial agents worsens the situation. O'Neill’s report predicts that with the current increasing trend of AMR, an estimated 10 million lives per year may be lost to AMR-related disease by 2050^3^. To avoid this worst-case scenario, it is necessary to develop new kinds of antimicrobial agents with chemical skeletons and/or antimicrobial mechanisms different from those of conventional drugs.

**Figure 1:**
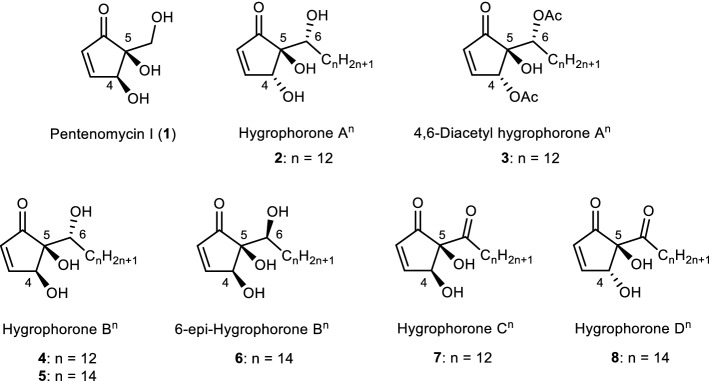
Chemical structures of pentenomycin I and representative hygrophorones.

Molecules with a cyclopentenone framework are Michael acceptors for various cellular nucleophiles due to their highly reactive α,β-unsaturated carbonyl moiety^4,5^. Thus, the five-membered carbon ring framework of cyclopentenone is often used as the core of building blocks to synthesize natural products and related compounds^6,7^. Additionally, highly oxygenated cyclopentenoids are known to be effective as promising antimicrobials^5^. Recently, we have reported the synthesis of pentenomycin I (**1**; Fig. 1), which was isolated from a cultured strain of *Streptomyces eurythermus*^8^, and its analogues^9^. In addition, we tested their antimicrobial activity and determined some of the structural factors that are important for antimicrobial activity. According to the results of this evaluation, their antimicrobial activity was moderate and unsuitable for pharmaceutical lead compounds. Therefore, advancing investigations into highly effective antimicrobial cyclopentenoids would undoubtedly be valuable for pharmaceutical development. Between 2004 and 2017, the group of Arnold has reported the isolation and structural elucidation of new cyclopentenoids, which they named hygrophorones, derived from various Hygrophorus species^10–14^. Isolated natural products are highly substituted 2-cyclopentenones with two hydroxy groups at asymmetric centers C-4 and C-5 and a hydrocarbon chain that contains an additional hydroxylated asymmetric center bonded at C-5. The structures of representative hygrophorones are shown in Fig. 1. Hygrophorones A–D (**2**–**8**) consist of a 2-cyclopenten-1-one skeleton substituted with hydroxy or acetoxy groups at the C-4 and C-5 positions and an oxidized long hydrocarbon chain attached to C-5. The structures and the stereochemistry of hygrophorones have been determined via extensive spectroscopic studies, including 2D NMR spectroscopy experiments. Furthermore, 4,6-diacetylhygrophorone A^12^ (**3**) showed potent antimicrobial activity against several bacterial species in the sub- to low-micromolar range (MIC = 0.25–8 µg/mL)^15^. Interestingly, **3**, which contains a long hydrocarbon chain, showed significantly higher antimicrobial activity than **1**^8,9^, which possesses an enone structure without an alkyl chain. Therefore, we hypothesize that other hygrophorones such as B type show high antibacterial activity. According to our survey of the literature, antimicrobial susceptibility tests of other hygrophorones toward bacterial-pathogen-caused human infectious diseases have not yet been reported, albeit in 2021 Westermann’s group disclosed the fungicidal activity of hygrophorone B^12^ (**4**) and its 6-deoxy analogue against plant pathogens^16^. Consequently, the development of efficient and flexible methods for the synthesis of the hygrophorone family, and the elucidation of their structure–activity relationship are desirable and useful from the viewpoint of medicinal/pharmaceutical chemistry.

**Scheme 1:**
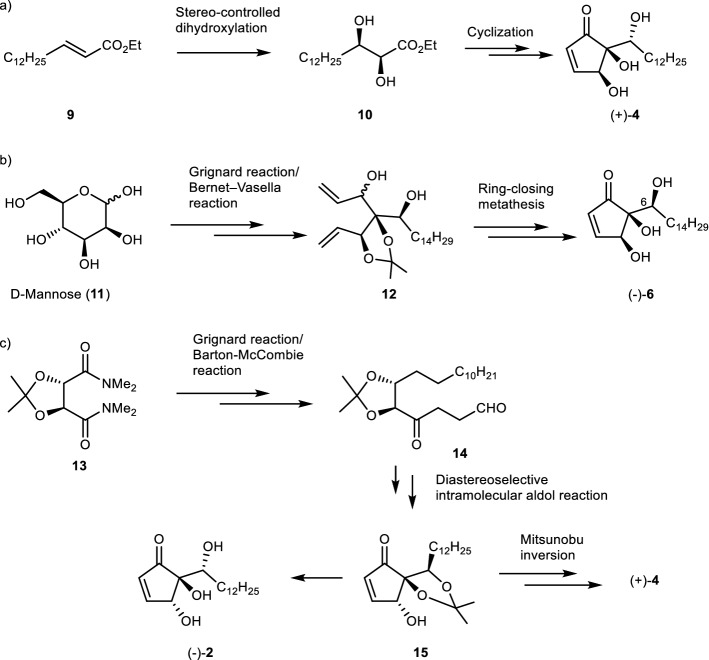
**a**: Key step in the synthesis of ( +)-hygrophorone B^12^ (**4**) by Westermann et al. **b**: Key step in the synthesis of (−)-6-*epi*-hygrophorone B^14^ (**6**) by Rao et al. **c**: Key step in the synthesis of (−)-hygrophorone A^12^ (**2**) and ( +)-**4** by Gholap et al.

Due to their unique structural features, hygrophorones have been drawing increasing attention and become a target for total synthesis. Numerous efforts have been devoted to the total synthesis of this class of natural products, and so far, three methods have been reported for the total synthesis of hygrophorone-B-type compounds and hygrophorone A^12^ (**2**)^17–19^. Westermann et al. reported the asymmetric total synthesis of hygrophorone A^12^ (**2**) and B^12^ (**4**) using a biomimetic strategy, in which the stereo-controlled dihydroxylation of fatty acid ester **9** is the key step (**9** → **10**). (+)-**2** and (−)-**4** were obtained using AD-mix-alpha, and the enantiomers (−)-**2** and (+)-**4** using AD-mix-beta (Scheme 1a)^17^. Rao et al. achieved the stereoselective synthesis of (−)-6-*epi*-hygrophorone B^14^ (**6**) from D-mannose (**11**) using a stereoselective Grignard reaction for the insertion of the hydrocarbon chain, the Bernet–Vasella protocol (**11** → **12**), and a ring-closing metathesis (Scheme 1b)^18^. More recently, the enantioselective syntheses of (−)-Hygrophorone A^12^ (**2**) and (+)-Hygrophorone B^12^ (**4)** from **13** has been achieved by Gholap et al. (Scheme 1c)^19^. The common intermediate **15** was prepared starting from **13** by a Grignard reaction for the insertion of the carbon chain, followed by a Barton–McCombie reaction (**13** → **14**), and finally a diastereoselective intramolecular aldol reaction (**14** → **15**) . The deprotection of acetonide in **15** produced (–)-Hygrophorone A^12^ (**2)**. In addition, Hygrophorone B^12^ (**4**) was obtained by epimerization at C-4 via the Mitsunobu inversion. All strategies are excellent methods for the stereoselective synthesis of the targeted hygrophorones. However, in order to synthesize hygrophorone analogues, an arbitrary carbon chain needs to be present from the beginning, or needs to be inserted at the earliest stage of the synthetic plan. In addition, there are so far no reports on the evaluation of the antimicrobial activity of such B-type hygrophorones with side chains of different length. Therefore, a new modular synthetic strategy for the preparation of a variety of hygrophorone analogues starting from a common intermediate would be useful to investigate the structure–activity relationship for hygrophorones. In this study, we focus on hygrophorone B^12^ (**4**) and we describe the enantioselective total synthesis of **4** and its analogues from cyclopentenone, as well as the evaluation of their antimicrobial activity.

## Results and discussion

**Scheme 2:**
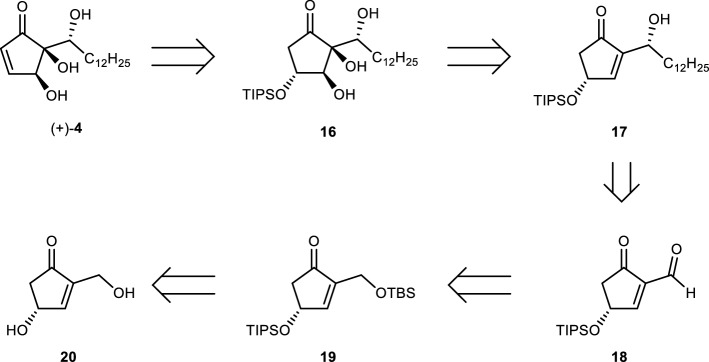
Retrosynthesis of ( +)-hygrophorone B^12^ (**4**). TBS = *tert*-butyldimethylsilyl; TIPS = triisopropylsilyl.

The outline of our synthetic plan is shown in Scheme 2. The most important component of our plan is the use of a key intermediate, i.e., formyl enone **18**, which is suitably functionalized and oxidized. The production of a wide variety of hygrophorone analogues from **18** could theoretically be achieved via a convenient and general method. It is assumed that a simple sequence could be proposed to insert any hydrocarbon chains and aromatic groups using organometallic reagents (**18** → **17**). After the insertion of the hydrocarbon chain, conversion into the hygrophorone skeleton would be accomplished by a stereoselective dihydroxylation followed by the formation of an enone moiety (**17** → **16** → **4**). The key intermediate **18** would be obtained from optically active cyclopentenone **20** via **19**.

**Scheme 3:**
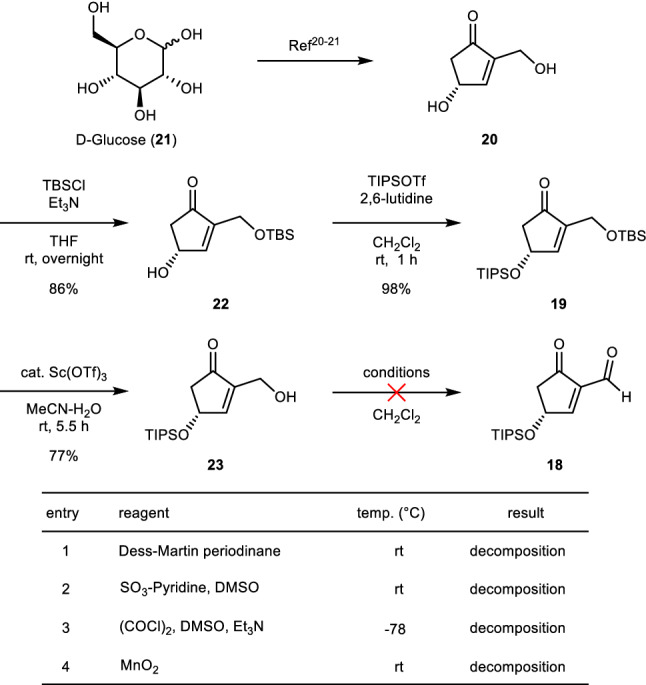
Toward the synthesis of aldehyde **18**. THF = tetrahydrofuran; TBS = *tert*-butyldimethylsilyl; TIPS = triisopropylsilyl; DMSO = dimethyl sulfoxide; rt: room temperature: temp. = temperature; for details, see the Supporting Information.

**Scheme 4:**
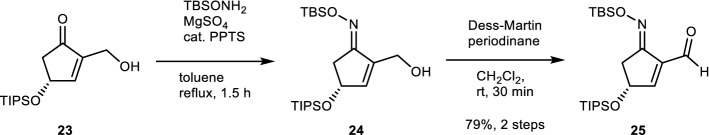
Synthesis of oxime **25**. TBS = *tert*-butyldimethylsilyl; TIPS = triisopropylsilyl; PPTS = pyridinium p-toluenesulfonate; rt: room temperature; for details, see the Supporting Information.

**Scheme 5.**
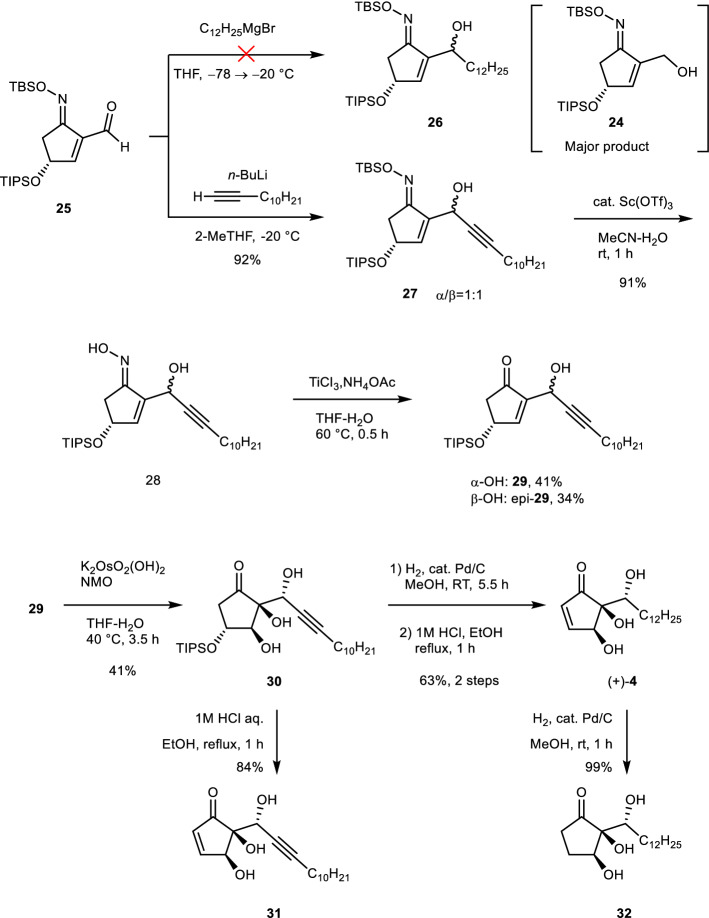
Synthesis of ( +)-hygrophorone B^12^ (**4**) and analogues **31**/**32**. THF = tetrahydrofuran; TBS = tert-butyldimethylsilyl; TIPS = triisopropylsilyl; NMO = *N*-methylmorpholine oxide; rt: room temperature; for details, see the Supporting Information.

The synthesis of key intermediate **18** is shown in Scheme 3. The starting material, cyclopentenone **20**, was obtained from D-glucose (**21**) according to published procedures^20,21^. First, **22** was synthesized from **20** by the selective protection of hydroxyl group by catalyst-free method using *tert*-butyldimethylsilylchloride (TBSCl) and triethylamine^9^, and then **19** was obtained by treatment with triisopropylsilyl trifluoromethanesulfonate (TIPSOTf) and 2,6-lutidine, followed by the regioselective desilylation of primary TBS under mild conditions using scandium (III) triflate as a catalyst (**19** → **23**). We next investigated the oxidation to obtain the desired formyl enone **18** (see the Table in Scheme 3). We examined oxidation methods to transform the primary hydroxyl group into a formyl group; however, **18** was not obtained under any of the attempted conditions. When the direct oxidation of the C2-hydroxymethyl group of **23** into a formyl group was attempted, the substrate decomposed, maybe due to the instability of the resulting enone with a formyl group at the C-2 position, which is highly electron-deficient. Therefore, it was crucial to control the electron density of the enone moiety by conversion of the ketone into an oxime. The electron-donating features of the oxime are able to reduce the electron deficiency of the enone moiety. *O*-(tert-butyldimethylsilyl)hydroxylamine (TBSONH_2_), which enabled easy access to oxime,“from the resulting *O*-silyl oxime” was used. The cleavage of oxime has been reported under a wide variety of conditions, and we expected that it would enable the conversion of the relatively hard enone–oxime into a ketone^22–27^. So, **23** was converted to **24** using TBSONH_2_, which was prepared from TBSCl and hydroxylamine^28^, with an acidic catalyst in the presence of anhydrous magnesium sulfate as water scavenger^29^. The resulting compound **24** was selectively obtained as *E* steroisomer avoiding the steric hindrance between the TBSO group and the hydroxymethyl group at the C-2 position of the enone. In the subsequent step, the Dess–Martin oxidation of the crude product **24** gave aldehyde **25** in 79% yield over two steps (Scheme 4). With key intermediate **25** in hand, we proceeded with the synthesis of (+)-**4** through the insertion of the hydrocarbon chain followed by the suitable ring modifications to obtain hygrophorone skeleton (Scheme 5). The coupling reaction of **25** with the hydrocarbon chain to construct the desired carbon skeleton of (+)-**4** was investigated intensively. Initial attempts with commercially available dodecylmagnesium bromide (C_12_H_25_MgBr) in tetrahydrofuran (THF) at − 78 to − 20 °C^30^ resulted in the formation of **24** as the major product, while compound **26** was barely produced. We assumed that this result is due to the reduction of the β-hydride via a six-membered-ring transition state between the formyl group of **25** and C_12_H_25_MgBr as a hydride source, similar to the Meerwein–Ponndorf–Verley (MPV) protocol (for details, see the Supporting Information, Scheme S1)^31–35^. To test this hypothesis, we used an unsaturated hydrocarbon chain, 1-dodecyne, without a hydride in the beta position. The organolithium obtained in situ from the treatment of 1-dodecyne with *n*-BuLi was allowed to react with intermediate **25** at − 40 °C to give **27** in moderate yield (57%) without forming **24**. Moreover, **27** was obtained in satisfactory yield (92%) as an inseparable diastereomeric mixture (α/β = 1:1) with regard to the C-6 position by using 2-methyl-THF, a less hygroscopic solvent than THF (for details, see the Supporting Information, Table S1). The scandium-catalyzed treatment of **27** under mild reaction conditions removed the TBS group to produce the oxime **28**, and a subsequent reductive deoximation by titanium (III) chloride in the presence of ammonium acetate^36^ gave **29** and *epi*-**29** as a mixture separable via column chromatography on silica gel. Compound **30** was prepared as a single stereoisomer from **29** by treatment with an osmium catalyst and *N*-methylmorpholine oxide at 70 °C; the formation of the osmate ester intermediate was stereoselective due to the steric hindrance of the TIPS group at the C-4 position. The catalytic hydrogenation of **30** over 10% Pd/C was performed in methanol at room temperature. Finally, the desilylation of the resulting intermediate and the subsequent elimination of the hydroxyl group to construct the enone moiety were efficiently achieved through treatment with 1 M aqueous HCl in ethanol at 90 °C^37^, affording the targeted (+)-hygrophorone B^12^ (**4**). The experimental evidences (^1^H and ^13^C NMR spectra and high-resolution mass spectrum) of synthetic (+)-**4** were identical to those of natural hygrophorone B^12^ (**4**). The optical rotation of synthetic (+)-4 ([α]_D_^25^ =  + 23.0; c = 0.10 in MeOH) matched that reported for natural **4** ([α]_D_^27^ =  + 20.7; c = 0.135, MeOH)^17^. Furthermore, we prepared two analogues of **4** starting from key intermediate **30**. Alkyne **31** was synthesized through direct treatment of **30** with 1 M aqueous HCl in ethanol at 90 °C, while the reduced analogue **32** was obtained by the Pd catalyzed hydrogenation of the endocyclic double bond of (+)-**4**.

The antimicrobial activity of the synthesized (+)-hygrophorone B^12^ (**4**) and its analogues **31** and **32** were evaluated to gauge their potency. Antimicrobial susceptibility testing was performed with the broth dilution method according to the Clinical and Laboratory Standards Institute (CLSI) 2020 guidelines^9,38^. Initially, the compounds were tested with six bacterial species (*Staphylococcus aureus, Enterococcus faecium, Escherichia coli, Klebsiella pneumoniae, Pseudomonas aeruginosa, and Acinetobacter baumannii*) that represent the most common pathogens in clinical settings. Gentamicin was used as a positive control in this assay, and the minimum inhibitory concentration (MIC) values are summarized in Table 1. (+)-**4** and **31** showed antimicrobial activity toward five of the bacterial species, excluding *P. aeruginosa*. In particular, they were significantly more effective against *E. faecium* than gentamicin, while the antimicrobial activity of **32** was remarkably lower against many species. These results indicate that the expression of antimicrobial activity depends on the α,β-unsaturated carbonyl framework in the hygrophorone B class of compounds. Moreover, the antimicrobial activities of (+)-**4** and **31** were investigated against six antimicrobial-resistant (AMR) bacteria isolates (methicillin-resistant *Staphylococcus aureus*, vancomycin-resistant *Enterococcus faecium*, carbapenem-resistant *Escherichia coli*, carbapenem-resistant Enterobacterales, multidrug-resistant *Pseudomonas aeruginosa*, and multidrug- resistant *Acinetobacter baumannii*) (Table 2). Intriguingly, (+)-**4** and **31** were notably effective in suppressing the growth of methicillin-resistant *S. aureus* (MRSA) and vancomycin-resistant *E. faecium* (VRE). Moreover, **31** with a carbon–carbon triple bond in its hydrocarbon chain suppressed the growth of multidrug-resistant *A. baumannii* (MDRA) compared to (+)-**4**. However, these compounds were not effective against carbapenem-resistant Enterobacterales (*E. coli* or *K. pneumoniae*) (CRE) or multidrug-resistant *P. aeruginosa* (MDRP). The aforementioned results show that hygrophorone-B-type compounds including **4** and **31** can be expected to exhibit higher antimicrobial activity against specific bacterial species, especially gram-positive bacteria. Detailed antimicrobial activity data, including MIC values for hygrophorone B^12^ (**4**) and its analogues **31**/**32** against bacterial pathogens causing human infectious diseases, have not been reported previously in the literature, while the MIC value of hygrophorone-A-type compound (**3**) has been reported elsewhere^15^. The results of our evaluation suggest that their chemical structure (e.g., the presence of triple bonds or enone moiety) have a substantial influence on the microbial activity of hygrophorones. Therefore, our strategy for a new synthesis of hygrophorone-B-type compounds from key intermediate **25** with a suitable side chain is useful. These findings demonstrated that hygrophorone B is an accessible lead compound for designing novel antimicrobial drugs.

**Table 1:** Antimicrobial evaluation of the synthesized hygrophorones (+)-**4**, **31**, and **32** using clinically relevant bacterial pathogens.

Organism^[a]^	MIC (µg/mL)^[b]^			
	( +)-4	31	32	Gem^[c]^
*S. aureus* ATCC 6358P	1	1	> 256	0.25
*E. faecium* ATCC 35,667	1	0.5	64	4
*E. coli* DH5 α	256	32	> 256	0.5
*K. pneumoniae* ATCC 10,031	32	8	> 256	0.5
*P. aeruginosa* PAO1	> 256	256	> 256	0.5
*A. baumannii* ATCC 17,978	32	16	> 256	16

Determined by CLSI broth microdilution methods.

^[a]^Measured concentration (0.125–256 µg/mL); *S. aureus*: *Staphylococcus aureus*; *E. faecium*: *Enterococcus faecium*; *E. coli: Escherichia coli; K. pneumoniae: Klebsiella pneumoniae; P. aeruginosa: Pseudomonas aeruginosa; A. baumannii: Acinetobacter baumannii.*

^[b]^MIC: Minimum inhibitory concentration.

^[c]^Gem: gentamicin. Measured concentration (0.125–16 µg/mL).

**Table 2:** Antimicrobial evaluation of synthesized (+)-**4** and **31** against antimicrobial-resistant (AMR) bacterial isolates.

Organism^[a]^	MIC (µg/mL)^[b]^		
	(+)-4	31	Gem^[c]^
MRSA MRY04-1385	1	1	> 16
VRE MRY05-0006	1	1	4
CRE (*E. coli*) MRY13-0331	> 256	> 256	> 16
CRE (*K. pneumoniae*) MRY12-0017	> 256	> 256	> 16
MDRP MRY09-1249	> 256	256	> 16
MDRA MRY12-0277	128	16	> 16

Determined by CLSI broth microdilution methods.

^[a]^Measured concentration (0.125–256 µg/mL). *MRSA*: methicillin-resistant *Staphylococcus aureus*; VRE: vancomycin-resistant *Enterococcus faecium*; CRE: carbapenem-resistant Enterobacterales (*Escherichia coli* or *Klebsiella pneumoniae*)*;* MDRP: multidrug-resistant *Pseudomonas aeruginosa;* MDRA: multidrug-resistant *Acinetobacter baumannii.*

^[b]^MIC: Minimum inhibitory concentration.

^[c]^Gem: gentamicin. Measured concentration (0.125–16 µg/mL).

## Conclusion

In conclusion, we have achieved the enantioselective total synthesis of (+)-hygrophorone B^12^ (**4**) and its analogues **31** and **32** in 4.5–4.8% overall yield over a linear sequence of 10–12 steps starting from cyclopentenone **20**, which can be obtained from d-glucose (**21**). The main advantage of our synthetic method is expected to contribute the provision of a wide variety of hygrophorone B analogues starting from the common key intermediate **25** via the insertion of various hydrocarbon chains or aryl groups. As ongoing research, we are investigating other side chains including aryl groups that can be introduced, and studying toward the synthesis of other analogues. The antimicrobial evaluation of (+)-**4** and its analogues revealed their potency, and the structure—activity relationship of hygrophorone-B-type compounds was disclosed. Moreover, the synthesized hygrophorones are highly effective in preventing the proliferation of chemical-sensitive bacteria and AMR bacteria (MRSA, VRE, and MDRA), especially gram-positive bacteria. These results can be expected to be useful for the design and development of new antimicrobial agents. Further studies concerning the synthesis of other hygrophorone B analogues and the evaluation of their antimicrobial activity, as well as an investigation of their side effects on human normal cells, are currently in progress in our group. In the near future, the bacterial intracellular target(s) that interact with hygrophorones will be revealed in detail, and we will provide accurate information on the mechanism of the expression of their activity.

## Methods

### Instruments

Optical rotations were recorded on an Anton Paar MCP-100. Nuclear magnetic resonance (NMR) spectra were recorded on a Bruker AVANCE–400 III and AVANCE-600 III spectrometer and calibrated using residual undeuterated solvent as an internal reference (CDCl_3_ at δ 7.26 ppm for ^1^H, δ 77.16 for ^13^C NMR). High–resolution mass spectrometry (HR–MS) was performed using a Bruker MicrOTOF–Q II–S1 using electrospray ionization (ESI) technique.

### General organic synthetic methods

Reactions were monitored by analytical thin layer chromatography (TLC) carried out on 0.25 mm E. Merck silica gel plates (60 F_254_). Visualization of the developed chromatogram was performed by UV absorbance and aqueous cerium ammonium molybdate. Flash chromatography was performed on Kanto Chemical silica gel 60 N (spherical, neutral, 40–50 µm) with the indicated solvent systems.

### Materials

Compound **20** (> 99% ee) and pyridinium *p*-toluenesulfonate (PPTS) stored in the freezer were used. trimethylamine (Et_3_N), 2,6-lutidine, anhydrous magnesium sulfate (MgSO_4_), ethylenediamine, ammonium acetate (NH_4_OAc), titanium(III) chloride (20% aqueous solution), potassium osmate(VI) dihydrate (K_2_OsO_2_(OH)_2_), palladium on carbon (10%, Pd/C), ethanol (EtOH), methanol (MeOH) and anhydrous solvents for organic synthesis, including CH_2_Cl_2_, tetrahydrofuran (THF) and acetonitrile (MeCN) were purchased from FUJIFILM Wako Pure Chemical Co. Triisopropylsilyl trifluoromethanesulfonate (TIPSOTf), scandium(III) trifluoromethanesulfonate (Sc(OTf)_3_) and 1-dodecyne were purchased from Tokyo Chemical Industry Co. *n*-butyllithium solution and methyllithium solution were purchased Kanto Chemical Co. *tert*-Butyldimethylsilyl chloride (TBSCl), dodecylmagnesium bromide solution (C_12_H_25_MgBr), *N*-methylmorpholine oxide (NMO), Gentamicin sulfate, anhydrous 2-methyl-THF for organic synthesis and phenylmagnesium bromide solution (PhMgBr) were purchased from Sigma-Aldrich Co. Gram–positive and Gram–negative bacterial reference strains, including *Stapylococcus aureus* ATCC 29,213, *Enterococcus faecium* ATCC 35,667, *Escherichia coli* DH5 α, *Klebsiella pneumoniae* ATCC 10,031, *Pseudomonas aeruginosa* PAO1, and *Acinetobacter baumannii* ATCC 17,978, *S. aureus* MRY04-1385, *E. faecium* MRY05-0006, *E. coli* MRY13-0331, *K. pneumoniae* MRY12-0017, *P. aeruginosa* MRY09-1249, *A. baumannii* MRY12-0277 were purchased from the American Type Culture Collection. Silica gel plates (60F–254) for thin layer chromatography were purchased from Merck. Silica gel 60 N (230–400 mesh) for flash chromatography was purchased from Kanto Chemical. All reagents were used without further purification. All reactions were carried out in flame–dried glassware under a nitrogen atmosphere with dry solvents. Unless stated otherwise, commercial grade reagents were used without further purification.

### Experimental procedures

Synthesis of **22.** TBSCl (5.24 g, 23.2 mmol) and Et_3_N (6.5 mL, 46.2 mmol) were added to a solution of cyclopentenone **20**^20,21^ (2.97 g, 23.2 mmol) in THF (77 mL). The reaction mixture was stirred at room temperature. After 24 h, the resulting mixture was quenched with saturated aqueous NH_4_Cl. The resulting mixture was extracted with EtOAc (2 × 100 mL). The combined extracts were washed with brine then dried with MgSO_4_. Concentration in vacuo afforded a residue, which was purified by column chromatography (hexane/EtOAc 3:1 → 1:1) to give **22** (4.86 g, 86%) as a colorless-pale yellow oil. TLC (Hexane:EtOAc, 1:3 v/v): R_*f*_ = 0.73; [α]_D_^20^ =  + 11.2 (c = 1.0 in CHCl_3_); ^1^H NMR (400 MHz, CDCl_3_): δ 0.080 (s, 3H), 0.085 (s, 3H), 0.92 (s, 9H), 1.91 (d, *J* = 5.2 Hz, 1H), 2.37 (dd, *J* = 2.0, 18.8 Hz, 1H), 2.86 (dd, *J* = 6.0, 18.8 Hz, 1H), 4.37–4.38 (m, 2H), 4.99 (brs, 1H), 7.37–7.38 (m, 1H) ppm; ^13^C NMR (100 MHz, CDCl_3_) δ − 5.35, − 5.32, 18.4, 26.0 (3C), 45.8, 58.0, 68.9, 148.4, 155.9, 204.8 ppm; HR–MS (ESI–TOF): m/z calcd. for C_12_H_23_O_3_Si ([M + H]^+^), 243.1411; found, 243.1415.

Synthesis of **19.** TIPSOTf (1.80 mL, 6.72 mmol) was added to a stirred solution of **22** (1.36 g, 5.61 mmol) and 2,6-lutidine (0.97 mL, 8.42 mmol) in CH_2_Cl_2_ (19 mL) at room temperature under argon atmosphere. After 1.5 h, the resulting mixture was quenched with saturated aqueous NH_4_Cl. The resulting mixture was extracted with EtOAc (2 × 100 mL), and the extracts were washed with brine then dried with MgSO_4_. Concentration under vacuum afforded a residue, which was purified by column chromatography (hexane/EtOAc 25:1 → 15:1) to give **19** (2.23 g, 99%) as a colorless oil. TLC (Hexane:EtOAc, 2:1 v/v): R_*f*_ = 0.90; [α]_D_^20^ =  + 17.5 (c = 1.0 in CHCl_3_); ^1^H NMR (400 MHz, CDCl_3_) δ 0.078 (s, 3H), 0.085 (s, 3H), 0.92 (s, 9H), 1.06–1.12 (m, 21H), 2.39 (dd, *J* = 2.0, 18.2 Hz, 1H), 2.81 (dd, *J* = 5.8, 18.2 Hz, 1H), 4.37 (t, *J* = 2.0 Hz, 2H), 4.99–5.03 (m, 2H), 7.32 (q, *J* = 2.2, 1H) ppm; ^13^C NMR (100 MHz, CDCl_3_) δ − 5.31, − 5.30, 12.2 (3C), 18.04 (4C), 18.05 (3C), 18.4 (2C), 25.9, 46.8, 58.0, 69.2, 147.2, 157.2, 205.1 ppm; HR–MS (ESI–TOF) : m/z C_21_H_43_O_3_Si_2_ ([M + H]^+^) calcd. for 399.2745, found 399.2741.

Synthesis of **23.** Sc(OTf)_3_ (173 mg, 0.352 mmol) and H_2_O (2.5 mL, 140 mmol) were added to a stirred solution of **19** (2.80 g, 7.04 mmol) in MeCN (70 mL) at room temperature, and the mixture was stirred at same temperature. After 5.5 h, the reaction mixture was quenched with saturated aqueous NaHCO_3_. The resulting mixture was extracted with EtOAc (2 × 100 mL). The combined extracts were washed with brine then dried with MgSO_4_. Concentration under vacuum afforded a residue, which was purified by column chromatography (hexane/EtOAc 4:1 → 2:1) to give **23** (1.53 g, 77%) as a colorless oil. TLC (Hexane:EtOAc, 4:1 v/v): R_*f*_ = 0.30; [α]_D_^20^ =  + 29.7 (c = 1.0 in CHCl_3_); ^1^H NMR (400 MHz, CDCl_3_) δ 1.05–1.16 (m, 21H), 2.11–2.15 (m, 1H), 2.39 (dd, J = 2.1, 18.3 Hz, 1H), 2.83 (dd, J = 6.0, 18.6 Hz, 1H), 4.34–4.45 (m, 2H), 5.02–5.05 (m, 1H), 7.31–7.32 (m, 1H) ppm; ^13^C NMR (100 MHz, CDCl_3_) δ 12.2 (3C), 18.03 (4C), 18.06 (2C), 46.5, 57.6, 69.3, 145.5, 157.7, 206.1 ppm; HR–MS (ESI–TOF) : m/z C_15_H_28_O_3_SiNa ([M + Na]^+^) calcd. for 307.1700, found 307.1697.

Synthesis of **25.** TBSONH_2_^28^ (775 mg, 5.26 mmol) and MgSO_4_ (169 mg, 1.40 mmol) were added to a stirred solution of **23** (1.0 g, 3.51 mmol) and PPTS (88 mg, 0.351 mmol) in toluene (3.5 mL) at room temperature, and the mixture was stirred at 100 °C. After 1.5 h, the reaction mixture was quenched with saturated aqueous NaHCO_3_. The resulting mixture was extracted with EtOAc (2 × 15 mL). The combined extracts were washed with brine then dried with MgSO_4_. Concentration under vacuum afforded a crude residue **24** (1.98 g), which was immediately used without any purification. crude residue **24**: ^1^H NMR (400 MHz, CDCl_3_) δ 0.15 (d, *J* = 13.5 Hz, 3H), 0.18 (d, *J* = 12.9 Hz, 3H), 0.93 (s, 9H), 1.07–1.14 (m, 21H), 2.51 (dd, *J* = 2.2, 18.4 Hz, 1H), 2.73 (brs,1H), 3.15 (dd, *J* = 6.5, 18.4 Hz, 1H), 4.37 (dd, *J* = 6.5, 12.7 Hz, 1H), 4.45 (dd, *J* = 5.2, 14.2 Hz, 1H), 4.95–4.98 (1H, m), 6.30–6.31 (m, 1H) ppm; ^13^C NMR (100 MHz, CDCl_3_) δ − 5.1 (2C), 12.2 (3C), 18.1 (2C), 18.4 (4C), 26.2 (4C), 37.4, 59.2, 72.6, 141.8, 141.9, 167.3 ppm.

Dess-Martin periodinane (DMP) (1.78 g, 4.21 mmol) was added to a solution of a crude residue **24** in CH_2_Cl_2_ (18 mL) at room temperature, and the mixture was stirred at same temperature. After 0.5 h, the reaction mixture was quenched with aqueous NaHCO_3_ and Na_2_S_2_O_3_. The resulting mixture was extracted with CHCl_3_ (2 × 30 mL). The combined extracts were washed with brine then dried with MgSO_4_. Concentration under vacuum afforded a residue, which was purified by column chromatography (hexane/EtOAc 30:1) to give **25** (1.14 g, 79%, 2 steps) as a pale-yellow oil. TLC (Hexane:EtOAc, 6:1 v/v): R_*f*_ = 0.65; [α]_D_^20^ =  + 113.1 (c = 0.51 in CHCl_3_); ^1^H NMR (400 MHz, CDCl_3_) δ 0.190 (s, 3H), 0.195 (s, 3H), 0.95 (s, 9H), 1.05–1.17 (m, 21H), 2.64 (dd, *J* = 2.9, 18.3 Hz, 1H), 3.26 (dd, *J* = 6.9, 18.5 Hz, 1H), 5.04–5.07 (m, 1H), 7.12–7.13 (d, *J* = 2.9 Hz 1H), 10.0 (s, 1H) ppm; ^13^C NMR (100 MHz, CDCl_3_) δ − 5.1 (2C), 12.2 (3C), 18.1 (4C), 18.4 (2C), 26.2 (4C), 37.6, 72.3, 138.9, 152.1, 163.8, 188.0 ppm; HR–MS (ESI–TOF) : m/z C_21_H_42_O_3_NSi_2_ ([M + H]^+^) calcd. for 412.2698, found 412.2703.

Synthesis of **27.** *n*-BuLi (1.59 M in n-hexane, 1.14 mL, 1.82 mmol) was dropwised to a solution of 1-dodecyne (0.39 mL, 1.82 mmol) in 2-methyl-THF (6 mL) at − 20 °C under argon atmosphere, and the mixture was stirred at same temperature. After 0.5 h, a solution of aldehyde **25** (500 mg, 1.21 mmol) in 2-methyl-THF (4 mL) was added to the resulting solution, and the mixture was stirred further an hour at same temperature. the reaction mixture was quenched with saturated aqueous NH_4_Cl. The resulting mixture was extracted with EtOAc (2 × 20 mL). The combined extracts were washed with brine then dried with MgSO_4_. Concentration under vacuum afforded a residue, which was purified by column chromatography (hexane/EtOAc 40:1) to give **27** (644 mg, 92%) as a pale-yellow syrup. TLC (Hexane:EtOAc, 6:1 v/v): R_*f*_ = 0.65; ^1^H NMR (400 MHz, CDCl_3_) δ 0.168–0.17 (6H), 0.195 (s, 3H), 0.87 (t, *J* = 6.9 Hz, 3H), 0.93–0.94 (9H), 1.05–1.14 (m, 21H), 1.26–1.43 (m, 14H), 1.48–1.51 (m, 2H), 2.21–2.28 (m, 2H), 2.54 (dd, *J* = 2.2, 18.4 Hz, 1H), 3.15–3.22 (m, 1H), 3.63 (d, *J* = 5.3 Hz, 0.5H), 3.82 (d, *J* = 5.3 Hz, 0.5H), 4.94–4.98 (m, 1H), 5.19–5.20 (m, 0.5H), 5.28–5.29 (m, 0.5H), 6.45–6.46 (m, 0.5H), 6.49–6.50 (m, 0.5H) ppm; ^13^C NMR (100 MHz, CDCl_3_) δ − 5.2, 12.1, 12.2, 14.3, 18.1, 18.4, 18.89, 18.9, 22.8, 26.18, 22.19, 28.7, 29.1, 29.3, 29.5, 29.7, 29.8, 32.0, 37.7, 37.8, 59.5, 59.6, 72.21, 72.24, 77.7, 77.9, 86.9, 87.0, 141.7, 141.8, 143.2, 143.6, 166.2, 166.5 ppm; HR–MS (ESI–TOF) : m/z C_33_H_64_O_3_NSi_2_ ([M + H]^+^) calcd. for 578.4419, found 578.4416.

Synthesis of **28.** Sc(OTf)_3_ (12.8 mg, 0.0259 mmol) and H_2_O (0.19 mL, 10.4 mmol) were added to a stirred solution of **27** (300 mg, 0.519 mmol) in MeCN (5.2 mL) at room temperature, and the mixture was stirred at same temperature. After an hour, the reaction mixture was quenched with saturated aqueous NaHCO_3_. The resulting mixture was extracted with CHCl_3_ (2 × 20 mL). The combined extracts were washed with brine then dried with MgSO_4_. Concentration under vacuum afforded a residue, which was purified by column chromatography (hexane/EtOAc 6:1 → 4:1) to give **28** (217 mg, 91%) as a pale-yellow syrup. TLC (Hexane:EtOAc, 4:1 v/v): R_*f*_ = 0.45; ^1^H NMR (400 MHz, CDCl_3_) δ 0.88 (t, *J* = 7.0 Hz, 3H), 1.05–1.17 (m, 21H), 1.22–1.39 (m, 14H), 1.49–1.54 (m, 2H), 2.23–2.28 (m, 2H), 2.59 (dd, *J* = 2.3, 18.3 Hz, 1H), 3.15 (brs, 0.5H), 3.18–3.25 (m, 1H), 3.36 (brs, 0.5H), 4.34–4.45 (m, 2H), 4.97–5.02 (m, 1H), 5.20 (s, 0.5H), 5.27 (s, 0.5H), 6.53–6.54 (m, 0.5H), 6.55–6.56 (m 0.5H), 7.18 (brs, 0.5H), 7.22 (brs, 0.5H) ppm; ^13^C NMR (100 Mz, CDCl_3_) δ 12.2, 14.3, 18.1, 18.9, 19.0, 22.8, 28.7, 29.1, 29.3, 29.5, 29.7, 29.8, 32.0, 37.3, 58.7, 58.9, 72.2, 72.3, 77.7, 77.9, 87.3, 141.5, 141.6, 162.6, 162.9 ppm; HR–MS (ESI–TOF) : m/z C_27_H_50_O_3_NSi ([M + H]^+^) calcd. for 464.3554, found 464.3547.

Synthesis of **29**/***epi*-29.** A solution of NH_4_OAc (830 mg, 10.8 mmol) in H_2_O (1.5 mL) and TiCl_3_ (20% aq., 2.66 mL, 3.45 mmol) were added to a stirred solution of **28** (200 mg, 0.431 mmol) in THF (5.1 mL) at room temperature, and the mixture was stirred at 60 °C. After 30 min, the reaction mixture was quenched with H_2_O. The resulting mixture was extracted with EtOAc (2 × 20 mL). The combined extracts were washed with saturated aqueous NaHCO_3_ and brine then dried with MgSO_4_. Concentration under vacuum afforded a residue, which was purified by column chromatography (hexane/EtOAc 13:1 → 10:1) to give **29** (39.9 mg, 41%) and ***epi*-29** (32.8 mg, 34%) as a pale-yellow viscous oil.

**29**; TLC (Hexane:EtOAc, 3:1 v/v): R_*f*_ = 0.55; [α]_D_^20^ =  + 12.0 (c = 1.0 in CHCl_3_); ^1^H NMR (400 MHz, CDCl_3_) δ 0.89 (t, *J* = 6.8 Hz, 3H), 1.05–1.19 (m, 21H), 1.27–1.40 (m, 14H), 1.50–1.57 (m, 2H), 2.26 (td, *J* = 2.0, 7.1 Hz, 2H), 2.46 (dd, *J* = 2.1, 18.3, 1H), 2.85–2.91 (m, 2H), 3.36 (brs, 0.5H), 5.03–5.06 (m, 1H), 5.21–5.22 (m, 1H), 7.43 (q, J = 3.4 Hz, 1H) ppm; ^13^C NMR (100 MHz, CDCl_3_) δ 12.2, 14.3, 18.02, 18.05, 18.9, 22.8, 28.6, 29.0, 29.3, 29.4, 29.6, 29.7, 32.0, 46.8, 57.8, 68.8, 87.7, 145.4, 168.6, 204.9 ppm; HR–MS (ESI–TOF) : m/z C_27_H_49_O_3_Si ([M + H]^+^) calcd. for 449.3445, found 449.3441.

***epi*-29**; TLC (Hexane:EtOAc, 3:1 v/v): R_*f*_ = 0.45; [α]_D_^20^ =  + 25.6 (c = 1.0 in CHCl_3_); ^1^H NMR (400 MHz, CDCl_3_) δ 0.87 (t, *J* = 7.0 Hz, 3H), 1.05–1.18 (m, 21H), 1.26–1.39 (m, 14H), 1.48–1.54 (m, 2H), 2.25 (dt, *J* = 2.0, 7.1 Hz, 2H), 2.43 (dd, *J* = 2.1, 18.3 Hz, 1H), 2.80 (dd, *J* = 5.8, 18.3 Hz, 1H), 3.05 (d, *J* = 4.3 Hz, 1H), 5.02–5.05 (m, 1H), 5.26 (brs, 1H), 7.43 (q, *J* = 3.5 Hz, 1H) ppm; ^13^C NMR (100 MHz, CDCl_3_) δ 12.1, 14.3, 18.02, 18.04, 18.9, 22.8, 28.6, 29.0, 29.3, 29.5, 29.7, 29.8, 32.0, 46.8, 58.1, 68.9, 87.7, 145.1, 168.9, 205.4 ppm; HR–MS (ESI–TOF) : m/z C_27_H_48_O_3_SiNa ([M + Na]^+^) calcd. for 471.3265, found 471.3251.

Synthesis of **30.** K_2_OsO_2_(OH)_4_ (2.3 mg, 6.13 µmol) and NMO (42.8 mg, 0.306 mmol) were added to a stirred solution of **29** (55 mg, 0.122 mmol) in THF–H_2_O (2.5 mL, 10:1) at room temperature, and the mixture was stirred at 40 °C. After 3.5 h, the reaction mixture was quenched with aqueous Na_2_S_2_O_3_ (10%). The resulting mixture was extracted with EtOAc (2 × 15 mL). The combined extracts were washed with brine (20 mL) then dried with MgSO_4_. Concentration under vacuum afforded a residue, which was purified by column chromatography (hexane/EtOAc 4:1) to give **30** (24.3 mg, 41%) as a pale-yellow oil. TLC (Hexane:EtOAc, 2:1 v/v): R_*f*_ = 0.40; [α]_D_^20^ = − 39.0 (c = 0.10 in CHCl_3_); ^1^H NMR (400 MHz, CDCl_3_) δ 0.88 (t, *J* = 7.1 Hz, 3H), 1.04–1.20 (m, 21H), 1.22–1.37 (m, 14H), 1.46–1.52 (m, 2H), 2.21 (dt, *J* = 2.0, 7.1 Hz, 2H), 2.44 (ddd, *J* = 1.0, 3.7, 20.6 Hz, 1H), 2.58 (brs, 1H), 2.85 (dd, *J* = 7.4, 19.6 Hz, 1H), 3.34 (brs, 1H), 3.72 (d, *J* = 10.6 Hz, 1H), 4.35 (d, *J* = 1.9 Hz), 1H), 4.45–4.48 (m, 1H), 4.55 (d, *J* = 9.8 Hz, 1H) ppm; ^13^C NMR (100 MHz, CDCl_3_) δ 12.0, 14.3, 179, 18.9, 22.8, 28.6, 29.0, 29.3, 29.5, 29.6, 29.7, 32.0, 44.5, 65.0, 71.5, 81.5, 89.3, 214.2 ppm; HR–MS (ESI–TOF) : m/z C_27_H_49_O_5_Si^−^ ([M-H]^−^) calcd. for 481.3355, found 481.3346.

Synthesis of (+)-Hygrophorone B^12^ (**4**). 10% Pd/C (1.7 mg, 10 w/w%) were added to a solution of **30** (16.8 mg, 0.0348 mmol) in MeOH (1.7 mL), and the reaction mixture was stirred under hydrogen atmosphere (balloon). After 5.5 h, the reaction mixture was filtered through a pad of celite and concentrated under reduced pressure. An obtained crude product (16.7 mg) which was used without any purification was dissolved in EtOH (1.2 mL). 1 M HCl (0.6 mL) was added to the stirred solution at room temperature, and the mixture was stirred at 90 °C. After 30 min, the reaction mixture was quenched with saturated aqueous NaHCO_3_. The resulting mixture was extracted with EtOAc (2 × 15 mL). The combined extracts were washed with brine (20 mL) then dried with MgSO_4_. Concentration under vacuum afforded a residue, which was purified by column chromatography (hexane/EtOAc 1:1) to give (+)-hygrophorone B^12^ (**4**) (6.7 mg, 63%, 2 steps) as a white solid. TLC (Hexane:EtOAc, 1:1 v/v): R_*f*_ = 0.30; [α]_D_^25^ =  + 23.0 (c = 0.10 in MeOH), {ref.^17^ [α]_D_^27^ =  + 20.7 (c = 0.135, MeOH)}, ^1^H and ^13^C NMR, and MS spectra were identical to those of natural ( +)-hygrophorone B^12^; ^1^H NMR (400 MHz, CDCl_3_) δ 0.88 (t, *J* = 6.9 Hz, 3H), 1.25–1.36 (m, 20H), 1.52–1.62 (m, 2H), 2.07 (d, *J* = 8.3, 1H), 2.94 (d, *J* = 7.3, 1H), 3.58 (s, 1H), 3.77 (t, *J* = 9.6 Hz, 1H), 4.72 (ddd, *J* = 1.4, 2.2, 7.3 Hz, 1H), 6.30 (dd, *J* = 1.3, 6.1 Hz, 1H), 7.64 (dd, *J* = 2.3, 6.1 Hz, 1H) ppm; ^13^C NMR (100 MHz, CDCl_3_) δ 14.3, 22.8, 26.3, 29.5, 29.6, 29.67, 29.7, 29.77, 29.8, 31.4, 32.1, 71.6, 73.5, 76.0, 133.7, 163.7, 207.5 ppm; HR–MS (ESI–TOF) : m/z C_18_H_31_O_4_^−^ ([M-H]^−^) calcd. for 311.2228, found 311.2219.

Synthesis of **31.** 1 M aqueous HCl (0.75 mL) was added to a stirred solution of **30** (11.4 mg, 0.0236 mmol) in ethanol (1.5 mL) at room temperature, and the mixture was stirred at 90 °C. After 30 min, the reaction mixture was quenched with saturated aqueous NaHCO_3_. The resulting mixture was extracted with EtOAc (2 × 15 mL). The combined extracts were washed with brine (20 mL) then dried with MgSO_4_. Concentration under vacuum afforded a residue, which was purified by column chromatography (hexane/EtOAc 4:3) to give **31** (6.1 mg, 84%) as a colorless amorphous. TLC (Hexane:EtOAc, 1:1 v/v): R_*f*_ = 0.2; [α]_D_^25^ = − 13.0 (c = 0.10 in CHCl_3_); ^1^H NMR (400 MHz, CDCl_3_) δ 0.88 (t, *J* = 7.0 Hz, 3H), 1.25–1.31 (m, 15H), 1.41–1.46 (m, 2H), 2.16 (dt, *J* = 2.0, 7.1 Hz, 1H), 2.88 (d, *J* = 7.9, 1H), 2.91 (d, *J* = 8.8, 1H), 3.83 (s, 1H), 4.55 (dt, *J* = 1.9, 8.8 Hz, 1H), 4.85 (dt, *J* = 1.7, 7.6 Hz, 1H), 6.31 (dd, *J* = 1.5, 6.1 Hz, 1H), 7.64 (dd, *J* = 2.2, 6.1 Hz, 1H) ppm; ^13^C NMR (100 MHz, CDCl_3_) δ 14.3, 18.7, 22.8, 28.5, 28.9, 29.2, 29.5, 29.6, 29.7, 32.0, 64.9, 71.9, 75.0, 76.0, 89.5, 133.4, 163.9, 206.1 ppm; HR–MS (ESI–TOF) : m/z C_18_H_27_O_4_^−^ ([M-H]^−^) calcd. for 307.1914, found 307.1915.

Synthesis of **32.** 10% Pd/C (0.27 mg, 10 w/w%) were added to a solution of (+)-**4** (2.7 mg, 8.64 µmol) in MeOH (1.0 mL), and the reaction mixture was stirred under hydrogen atmosphere (balloon). After an hour, the reaction mixture was filtered through a pad of celite and concentrated under reduced pressure. The residue was purified by column chromatography (hexane/EtOAc 1:1) to give to **32** (2.7 mg, 99%) as a white solid. TLC (Hexane:EtOAc, 1:3 v/v): Rf. = 0.6; [α]_D_^25^ = − 26.0 (c = 0.10 in CHCl_3_); ^1^H NMR (400 MHz, CDCl_3_) δ 0.88 (t, *J* = 6.8 Hz, 3H), 1.25–1.42 (m, 21H), 1.51–1.53 (m, 1H), 1.87 (d, *J* = 9.7 Hz, 1H), 2.04–2.12 (m, 1H), 2.23–2.54 (m, 4H), 3.13 (s, 1H), 3.60–3.65 (m, 1H), 4.40–4.41 (m, 1H) ppm; ^13^C NMR (150 MHz, CDCl_3_) δ 14.3, 22.8, 25.4, 26.0, 29.5, 29.56, 29.66, 29.71, 29.77 (2C), 29.8, 31.8, 32.1, 32.7, 71.3, 72.0, 83.4, 217.3 ppm; HR–MS (ESI–TOF) : m/z C_18_H_33_O_4_^−^ ([M-H]^−^) calcd. for 313.2384, found 313.2354.

## Supplementary Information

Supplementary Information.

## Data Availability

The datasets used and/or analyzed during the current study available from the corresponding author on reasonable request.

## References

[1] Fischbach MA, Walsh CT (2009). Antibiotics for emerging pathogens. Science.

[2] Zhu Y-G, Zhao Y, Li B, Huang C-L, Zhang S-Y, Yu S, Chen Y-S, Zhang T, Gillings MR, Su J-Q (2017). Continental-scale pollution of estuaries with antibiotic resistance genes. Nat. Microbiol..

[3] O’Neill J (2016). Tackling drug-resistant infections globally: Final report and recommendations. Rev. Antimicrob Resist..

[4] Bickley JF, Ciucci A, Evans P, Roberts SM, Ross N, Santoro MG (2004). Reactions of some cyclopentenones with selected cysteine derivatives and biological activities of the product thioethers. Bioorg. Med. Chem..

[5] Arjona O, Gómez AM, López JC, Plumet J (2007). Synthesis and conformational and biological aspects of carbasugars. Chem. Rev..

[6] Roche SP, Aitken DJ (2010). Chemistry of 4-Hydroxy-2-cyclopentenone Derivatives. Eur. J. Org. Chem..

[7] Simeonov SP, Nunes JPM, Guerra K, Kurteva VB, Afonso CAM (2016). Synthesis of chiral cyclopentenones. Chem. Rev..

[8] Umino K, Furumai T, Matsuzawa N, Awataguchi Y, Ito Y, Okuda T (1973). Studies on pentenomycins. I. J. Antibiot..

[9] Kamishima T, Suzuki M, Aoyagi S, Watanabe T, Koseki Y, Kasai H (2019). A facile synthesis of (+)/(−)-pentenomycin I and analogs, and their antimicrobial evaluation. Tetrahedron Lett..

[10] Lübken T, Schmidt J, Porzel A, Arnold N, Wessjohann L (2004). Hygrophorones A-G: Fungicidal cyclopentenones from *Hygrophorus* species (Basidiomycetes). Phytochemistry.

[11] Otto A, Porzel A, Westermann B, Brandt W, Wessjohann L, Arnold N (2017). Structural and stereochemical elucidation of new hygrophorones from *Hygrophorus abieticola* (Basidiomycetes). Tetrahedron.

[12] Lübken T, Arnold N, Wessjohann L, Böttcher C, Schmidt J (2006). Analysis of fungal cyclopentenone derivatives from Hygrophorus spp. by liquid chromatography/electrospray-tandem mass spectrometry. J. Mass Spectrom..

[13] Teichert A, Lübken T, Schmidt J, Porzel A, Arnold N, Wessjohann LA (2005). Unusual bioactive 4-oxo-2-alkenoic fatty acids from Hygrophorus eburneus. Z. Naturforsch..

[14] Otto A, Porzel A, Schmidt J, Brandt W, Wessjohann L, Arnold N (2016). Structure and absolute configuration of pseudohygrophorones A^12^ and B^12^, alkyl cyclohexenone derivatives from *Hygrophorus abieticola* (Basidiomycetes). J. Nat. Prod..

[15] PhD thesis, Lübken, T. Martin-Luther-Universität Halle-Wittenberg, Hygrophorone Neue antifungische Cyclopentenonderivate aus Hygrophorus-Arten (Basidiomycetes). (2006).

[16] Ditfe T, Bette E, Sultani HN, Otto A, Wessjohann LA, Arnold N, Westermann B (2021). Synthesis and biological evaluation of highly potent fungicidal deoxy-hygrophorones. Eur. J. Org. Chem..

[17] Bette E, Otto A, Dräger T, Merzweiler K, Arnold N, Wessjohann L, Westermann B (2015). Isolation and asymmetric total synthesis of fungal secondary metabolite hygrophorone B^12^. Eur. J. Org. Chem..

[18] Surender B, Vinaykumar A, Kumar BS, Vemulapalli SPB, Rao BV (2018). Stereoselective total synthesis of (–)-6-*epi*-hygrophorone B^14^ and (–)-4-*epi*-hygrophorone D^14^. ChemistrySelect.

[19] Das S, Dalal A, Gholap SL (2021). Stereoselective total syntheses of (−)-hygrophorone A^12^, 4-O-acetyl-hygrophorone A^12^ and (+)-hygrophorone B^12^. Org. Biomol. Chem..

[20] Kamishima T, Nonaka T, Watanabe T, Koseki Y, Kasai H (2018). One-step conversion to a disubstituted cyclopentenone from 2-deoxy-D-glucose and application to synthesis of prostaglandin E_1_ methyl ester. Bull. Chem. Soc. Jpn..

[21] Koseki Y, Watanabe T, Kamishima T, Kwon E, Kasai H (2019). Formation of five-membered carbocycles from D-glucose: A concise synthesis of 4-hydroxy-2-(hydroxymethyl)cyclopentenone. Bull. Chem. Soc. Jpn..

[22] Corey EJ, Niimura K, Konishi Y, Hashimoto S, Hamada Y (1986). A new synthetic route to prostaglandins. Tetrahedron Lett..

[23] Timms GH, Wildsmith E (1971). The reduction of oximes with tervalent titanium, a mild deoximation procedure and the partial synthesis of erythromycylamine. Tetrahedron Lett..

[24] Shaabani A, Hezarkhania Z, Badali E (2015). Wool supported manganese dioxide nano-scale dispersion: A biopolymer based catalyst for the aerobic oxidation of organic compounds. RSC Adv..

[25] Jie Z, Rammoorty V, Fischer B (2002). Diethyl chlorophosphite: A versatile reagent. J. Org. Chem..

[26] DePuy CH, Ponder BW (1959). Levulinic acid as a reagent for the hydrolysis of oximes and 2,4-dinitrophenylhydrazones. J. Am. Chem. Soc..

[27] Yamaguchi J, Toyoshima M, Shoji M, Kakeya H, Osada H, Hayashi Y (2006). Concise enantio- and diastereoselective total syntheses of fumagillol, RK-805, FR65814, ovalicin, and 5-demethylovalicin. Angew. Chem. Int. Ed..

[28] Hughes JME, Gleason JL (2017). A concise enantioselective total synthesis of (−)-Virosaine|A. Angew. Chem. Int. Ed..

[29] Shibata N, Tsuchiya T, Hashimoto Y, Morita N, Ban S, Tamura O (2017). Thiyl radical-mediated cyclization of ω-alkynyl *O*-tert-butyldiphenylsilyloximes. Org. Biomol. Chem..

[30] Wang Z-M, Qian X-H, Zhou W-S (1990). Stereoselective synthesis of (-)-(5R,6S)-6-acetoxy-5-hexadecanolide, the mosquito oviposition attractant pheromone. Tetrahedron.

[31] Miller J, Gregoriou G, Mosher HS (1961). Relative rates of Grignard addition and reduction reactions^1-3^. J. Am. Chem. Soc..

[32] Rachwal S, Kawalek B, Gorecki T, Milart P (1990). Spiroferrocenophanes II. Grignard addition and reduction reactions of spiro[5]ferrocenophane-3,1’-cyclohexane-1,5-dione. J. Organomet. Chem..

[33] Meerwein H, Schmidt R (1925). Ein neues Verfahren zur Reduktion von Aldehyden und Ketonen. Justus Liebigs Ann. Chem..

[34] Ponndorf WD (1926). reversible austausch der oxydationsstufen zwischen aldehyden oder ketonen einerseits und primären oder sekundären alkoholen anderseits. Angew. Chem..

[35] Verley A (1925). Exchange of functional groups between two molecules. Bull. Soc. Chim. Fr..

[36] Baraldi PG, Barco A, Benetti S, Pollini GP, Simoni D, Zanirato V (1987). Stereospecific nitromethane conjugate addition to 4-oxygenated-2-substituted-cyclo pent-2-enones: A simple approach to prostaglandins. Tetrahedron.

[37] Cunico RF, Bedell L (1980). The triisopropylsilyl group as a hydroxyl-protecting function. J. Org. Chem..

[38] CLSI. Performance Standards for Antimicrobial Susceptibility Testing. 30th ed. CLSI guideline M100 (2020).

