# Supplementary material for: Total synthesis and antimicrobial evaluation of (+)-hygrophorone B12 and its analogues
Source: Sci Rep. 2022 May 6;12:7471. doi: 10.1038/s41598-022-11608-8 (PMC9076842; doi:10.1038/s41598-022-11608-8)
Supplement: Supplementary file 1 — Supplementary Information. [file 41598_2022_11608_MOESM1_ESM.pdf]

Supplementary Information

Total Synthesis and Antimicrobial Evaluation of (+)-Hygrophorone B[12] and its Analogues

Takaaki Kamishima,[1*] Masato Suzuki,[2] Koichi Narita,[3] Yoshitaka Koseki,[4] Toshiyuki Nonaka,[1] Hirotaka Nakatsuji,[1] Hideo Hattori,[5] and Hitoshi Kasai[4*]

1) East Tokyo Laboratory, Genesis Research Institute, Inc. 717-86 Futamata, Ichikawa, Chiba 272-0001, Japan

2) Antimicrobial Resistance Research Center, National Institute of Infectious Diseases 4-2-1 Aobamachi, Higashimurayama, Tokyo 189-0002, Japan

3) Faculty of Pharmaceutical Sciences, Tohoku Medical and Pharmaceutical University 4-4-1 Komatsushima, Aoba-ku, Sendai, Miyagi 981-8558, Japan

4) Institute of Multidisciplinary Research for Advanced Materials, Tohoku University 2-1-1 Katahira, Aoba-ku, Sendai, Miyagi 980-8577, Japan

5) Fromseeds Corporation, 6-6-40 Aramaki, Aoba-ku, Sendai, Miyagi, 980-0845, Japan.

Takaaki Kamishima
E-mail: t.kamishima@konpon.co.jp
Phone: +81-22-217-5101
FAX: +81-22-217-5614

Hitoshi Kasai
E-mail: kasai@tohoku.ac.jp
Phone: +81-22-217-5612
FAX: +81-22-217-5614

Table of Contents

Insertion of hydrocarbon chain or aryl group into **24** leading to hygrophorone precursor S2

Reaction mechanism of β-hydride reduction ········ S3

^{1}H and ^{13}C NMR Spectra of experimental procedures ········ S4

Table S1. Insertion of hydrocarbon chain or aryl group into **24** leading to hygrophorone precursor[a]

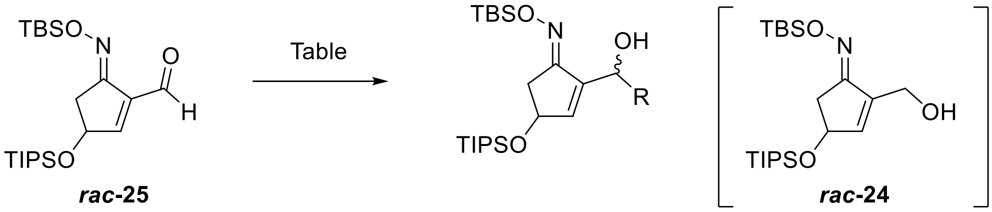

Entry	Conditions[b]	Results / Yields[c]
1	$C_{12}H_{25}MgBr$ (2 eq.), THF, −78°C → −20°C	R = $C_{12}H_{25}$ **S2**, trace amount<br>**24**, 78%
2	$C_{12}H_{25}MgBr$ (2 eq.), $CeCl_3$ (2.2 eq.),<br>THF, −78°C → −20°C	No reaction
3	1-dodecyne (2 eq.), n-BuLi (1.59M, 2 eq.)<br>THF, −78°C → −40°C	R = $C_{12}H_{21}$ **27**, 57%<br>**24**, not detected
4	1-dodecyne (2 eq.), n-BuLi (1.59M, 2 eq.)<br>2-MeTHF, −78°C → −20°C	R = $C_{12}H_{21}$ **27**, 85%<br>**24**, not detected
5	1-dodecyne (1.5 eq.), n-BuLi (1.59M, 1.5 eq.)<br>2-MeTHF, −20°C	R = $C_{12}H_{21}$ **27**, 92%<br>**24**, not detected
6	PhMgBr (1.5 eq.), 2-MeTHF, −20°C	R = Ph **S2**, 75%<br>**24**, not detected
7	MeLi (1.5 eq.), 2-MeTHF, −20°C	R = Me **S3**, 60%<br>**24**, not detected

[a] All reactions were carried out using a racemic compound of **25**; [b] All reactions were carried out under argon atmosphere. [c] All yields refer to be purified, isolated products.

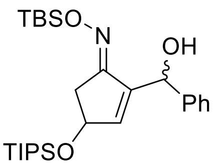

S2

S2: ^{1}H NMR (400 MHz, $CDCl_3$) δ 0.12 (s, 3.5H), 0.15 (d, *J* =1.4 Hz, 2.5H), 0.88 (s, 5.5H), 0.91 (s, 3.5H), 1.02-1.08 (m, 21H), 2.49-2.55 (m, 1H), 3.10-3.18 (m, 1H), 3.73 (d, *J* =5.4 Hz, 0.6H), 3.99 (d, *J* =4.6 Hz, 0.4H), 4.90-4.96 (m, 1H), 5.57 (d, *J* =51 Hz, 0.6H), 5.59 (d, *J* =4.3 Hz, 0.4H), 6.04-6.05 (m, 0.4H), 6.19-6.20 (m, 0.6H), 7.26-7.42 (m, 5H).

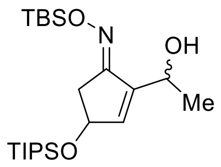

S3

S3: ^{1}H NMR (400 MHz, $CDCl_3$) δ 0.166 (s, 3H), 0.169 (s, 3H), 0.94 (s, 9H), 1.05-1.14 (m, 21H), 1.43 (t, *J* = 6.4 Hz, 1H), 2.48-2.53 (m, 1H), 3.11-3.19 (m, 1H), 3.32 (d, J =4.9 Hz, 0.5H), 3.57 (d, J = 3.6 Hz, 0.5H), 4.57-4.67 (m, 1H), 4.92-4.95 (m, 1H), 6.23-6.25 (m, 1H).

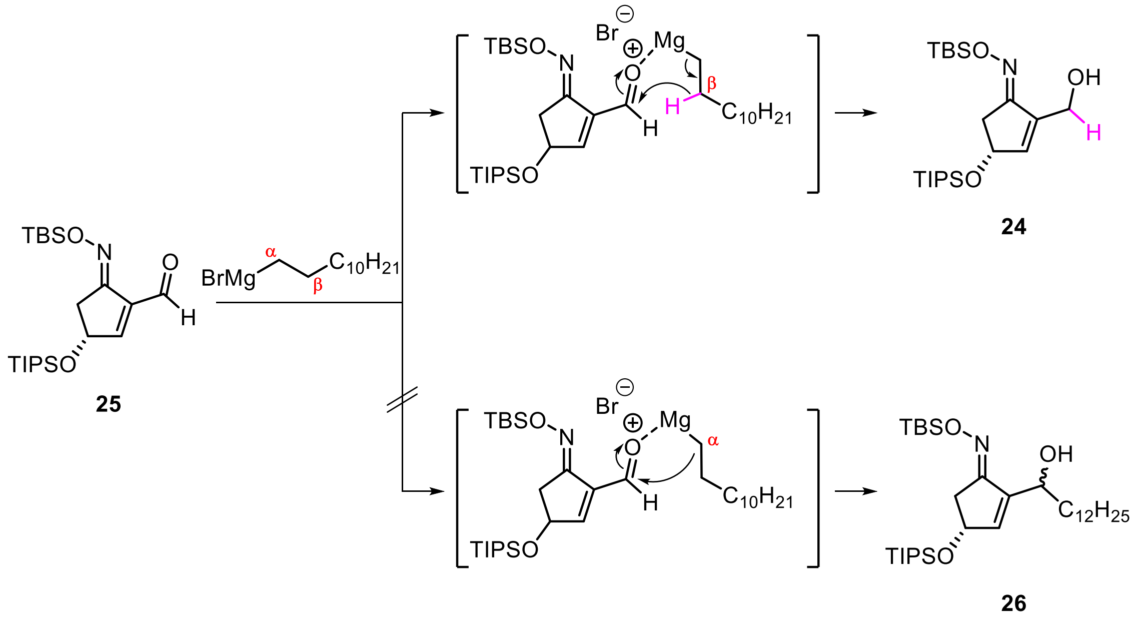

Scheme S1. Proposed reaction mechanism of β-hydride reduction that occurs by reacting **25** with a Grignard reagent of saturated hydrocarbon chain.

^{1}H and ^{13}C NMR Spectra

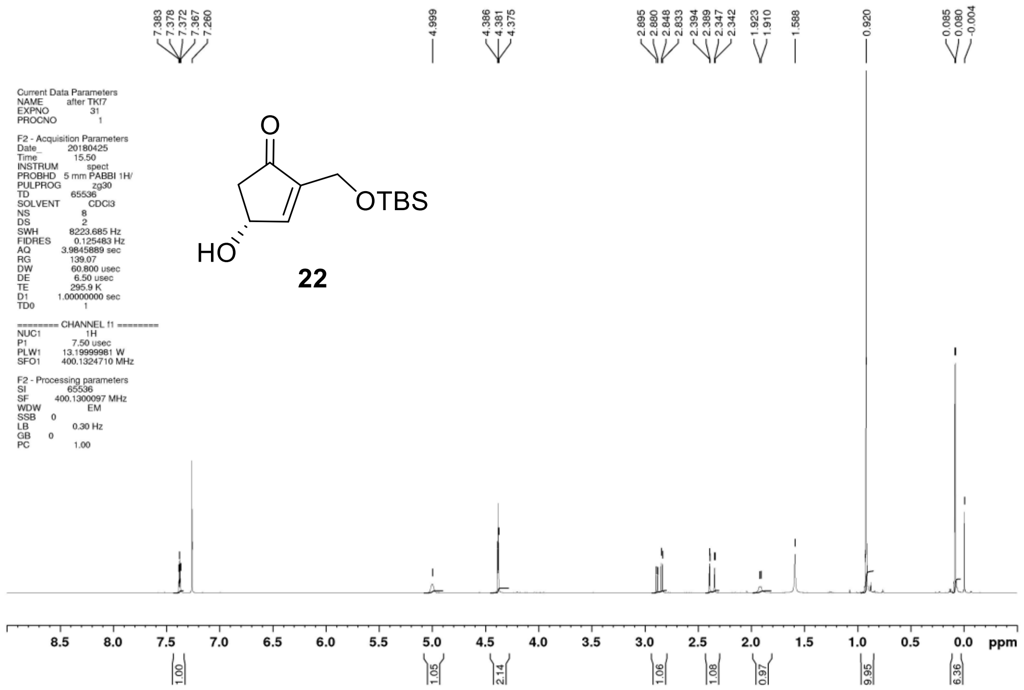

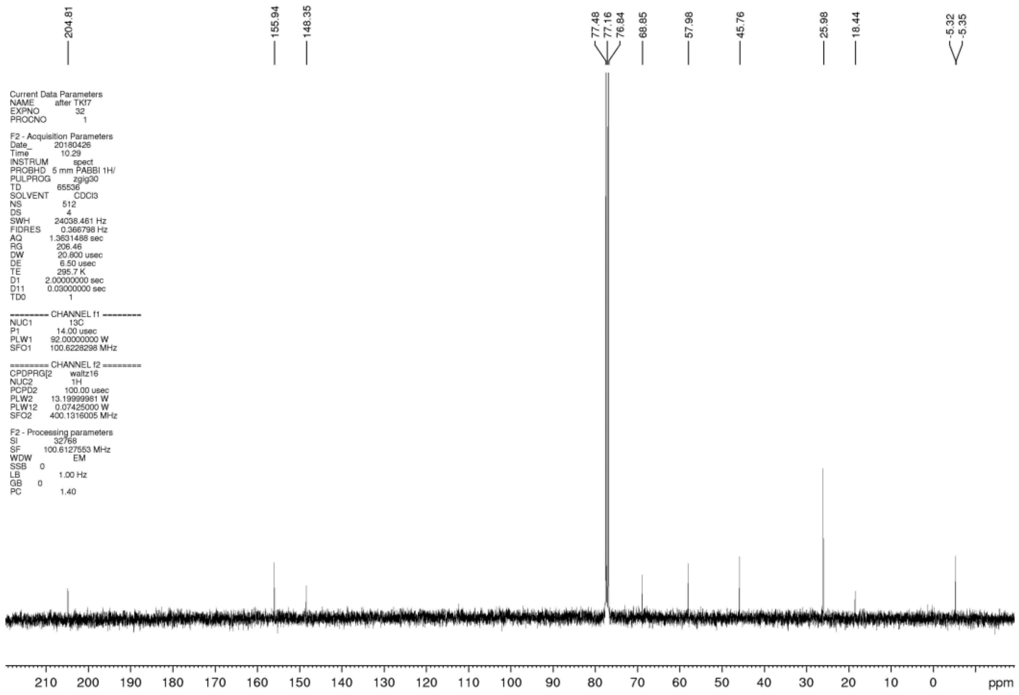

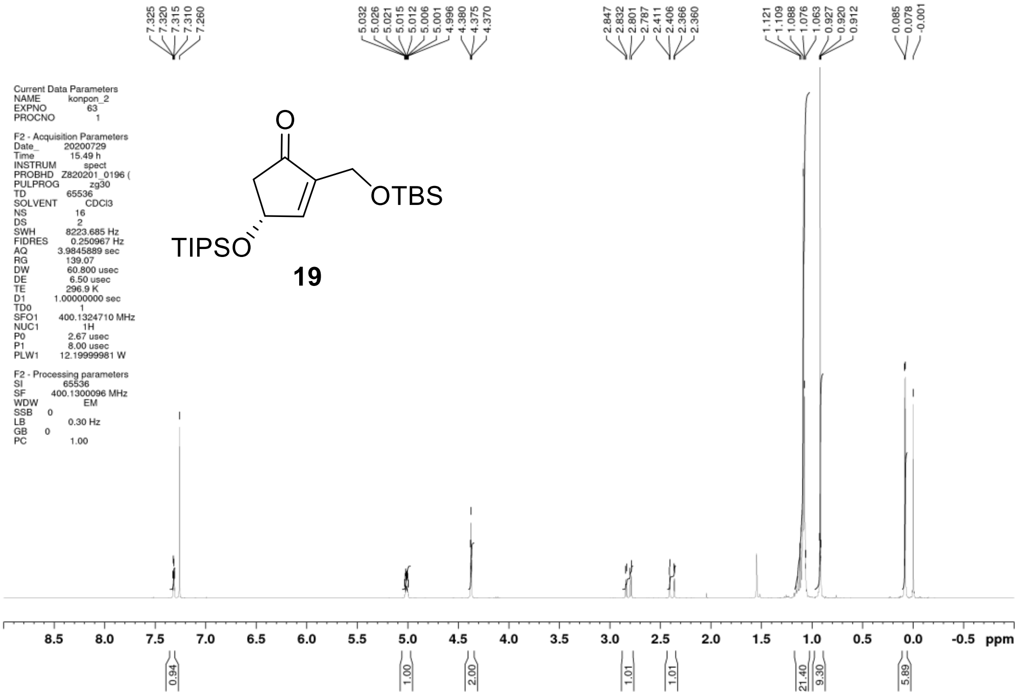
7.325
7.320
7.315
7.310
7.260
5.032
5.026
5.021
5.015
5.012
5.006
5.001
4.996
4.380
4.375
4.370
2.847
2.832
2.801
2.787
2.411
2.406
2.366
2.360
1.121
1.109
1.088
1.076
1.063
0.927
0.920
0.912
0.085
0.078
-0.001
Current Data Parameters
NAME konpon_2
EXPNO 63
PROCNO 1
F2 - Acquisition Parameters
Date_ 20200729
Time 15.49 h
INSTRUM spect
PROBHD Z820201_0196 (
PULPROG zg30
TD 65536
SOLVENT CDCl3
NS 16
DS 2
SWH 8223.685 Hz
FIDRES 0.250967 Hz
AQ 3.9845889 sec
RG 139.07
DW 60.800 usec
DE 6.50 usec
TE 296.9 K
D1 1.00000000 sec
TD0 1
SFO1 400.1324710 MHz
NUC1 1H
P0 2.67 usec
P1 8.00 usec
PLW1 12.19999981 W
F2 - Processing parameters
SI 65536
SF 400.1300096 MHz
WDW EM
SSB 0
LB 0.30 Hz
GB 0
PC 1.00
O
OTBS
TIPSO
19
8.5
8.0
7.5
7.0
6.5
6.0
5.5
5.0
4.5
4.0
3.5
3.0
2.5
2.0
1.5
1.0
0.5
0.0
-0.5
ppm
0.94
1.00
2.00
1.01
1.01
21.40
9.30
5.89

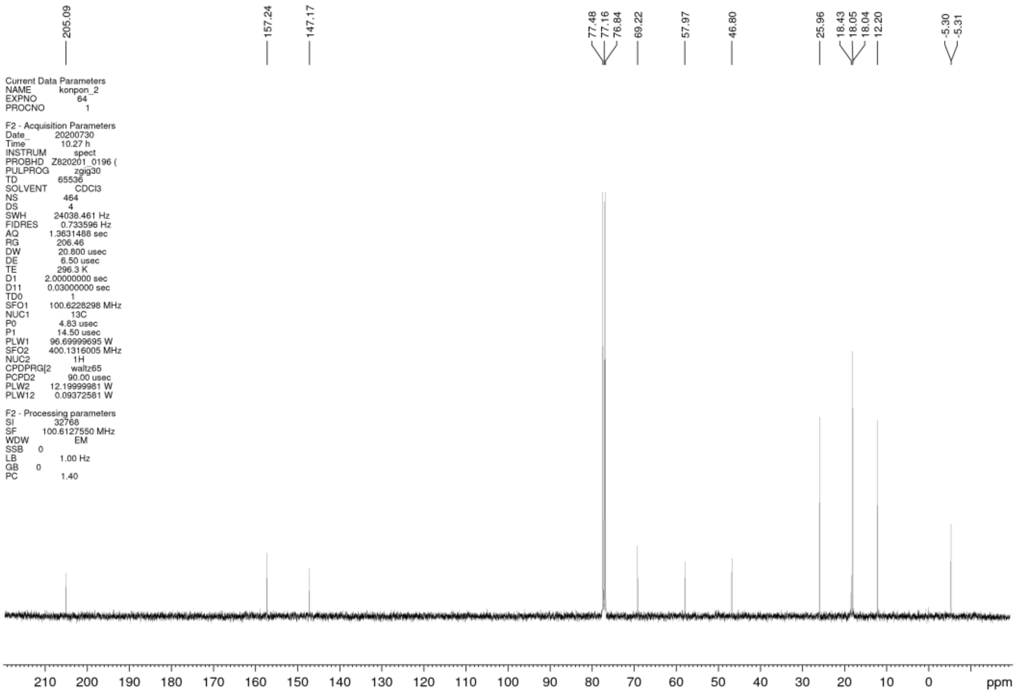
205.09
157.24
147.17
77.48
77.16
76.84
69.22
57.97
46.80
25.96
18.43
18.05
18.04
12.20
-5.30
-5.31
Current Data Parameters
NAME konpon_2
EXPNO 64
PROCNO 1
F2 - Acquisition Parameters
Date_ 20200730
Time 10.27 h
INSTRUM spect
PROBHD Z820201_0196 (
PULPROG zgig30
TD 65536
SOLVENT CDCl3
NS 464
DS 4
SWH 24038.461 Hz
FIDRES 0.733596 Hz
AQ 1.3631488 sec
RG 206.46
DW 20.800 usec
DE 6.50 usec
TE 296.3 K
D1 2.00000000 sec
D11 0.03000000 sec
TD0 1
SFO1 100.6228298 MHz
NUC1 13C
P0 4.83 usec
P1 14.50 usec
PLW1 96.69999695 W
SFO2 400.1316005 MHz
NUC2 1H
CPDPRG[2 waltz65
PCPD2 90.00 usec
PLW2 12.19999981 W
PLW12 0.09372581 W
F2 - Processing parameters
SI 32768
SF 100.6127550 MHz
WDW EM
SSB 0
LB 1.00 Hz
GB 0
PC 1.40
210
200
190
180
170
160
150
140
130
120
110
100
90
80
70
60
50
40
30
20
10
0
ppm

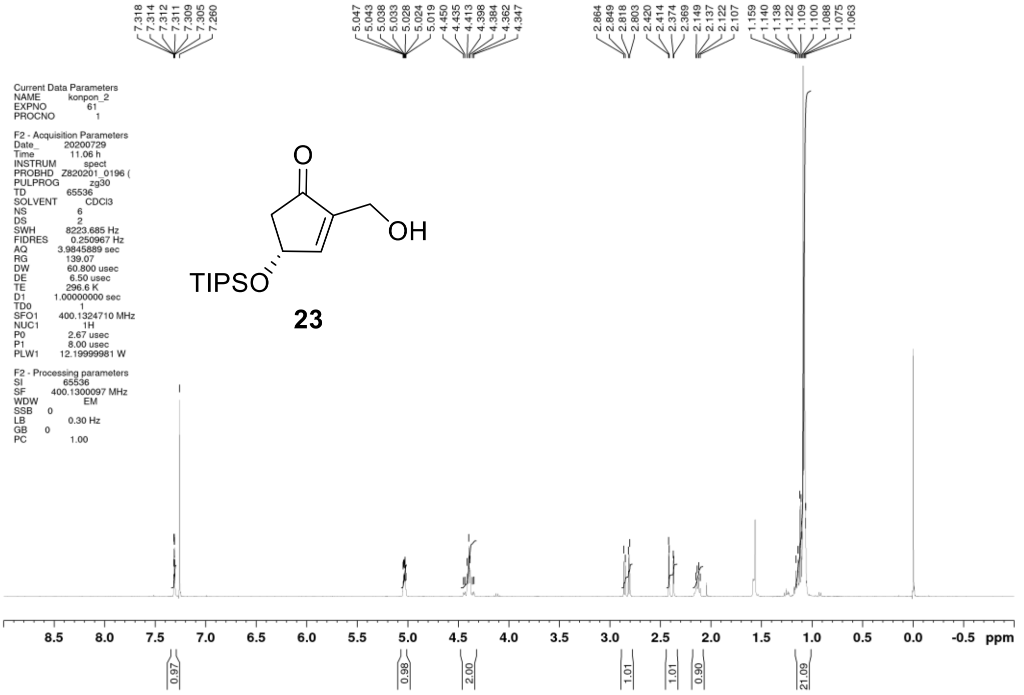
7.318
7.314
7.312
7.311
7.309
7.305
7.260
5.047
5.043
5.038
5.033
5.028
5.024
5.019
4.450
4.435
4.413
4.398
4.384
4.362
4.347
2.864
2.849
2.818
2.803
2.420
2.414
2.374
2.369
2.149
2.137
2.122
2.107
1.159
1.140
1.138
1.122
1.109
1.100
1.088
1.075
1.063
Current Data Parameters
NAME konpon_2
EXPNO 61
PROCNO 1
F2 - Acquisition Parameters
Date_ 20200729
Time 11.06 h
INSTRUM spect
PROBHD Z820201_0196 (
PULPROG zg30
TD 65536
SOLVENT CDCl3
NS 6
DS 2
SWH 8223.685 Hz
FIDRES 0.250967 Hz
AQ 3.9845889 sec
RG 139.07
DW 60.800 usec
DE 6.50 usec
TE 296.6 K
D1 1.00000000 sec
TD0 1
SFO1 400.1324710 MHz
NUC1 1H
P0 2.67 usec
P1 8.00 usec
PLW1 12.19999981 W
F2 - Processing parameters
SI 65536
SF 400.1300097 MHz
WDW EM
SSB 0
LB 0.30 Hz
GB 0
PC 1.00
O
OH
TIPSO
23
8.5
8.0
7.5
7.0
6.5
6.0
5.5
5.0
4.5
4.0
3.5
3.0
2.5
2.0
1.5
1.0
0.5
0.0
-0.5
ppm
0.97
0.98
2.00
1.01
1.01
0.90
21.09

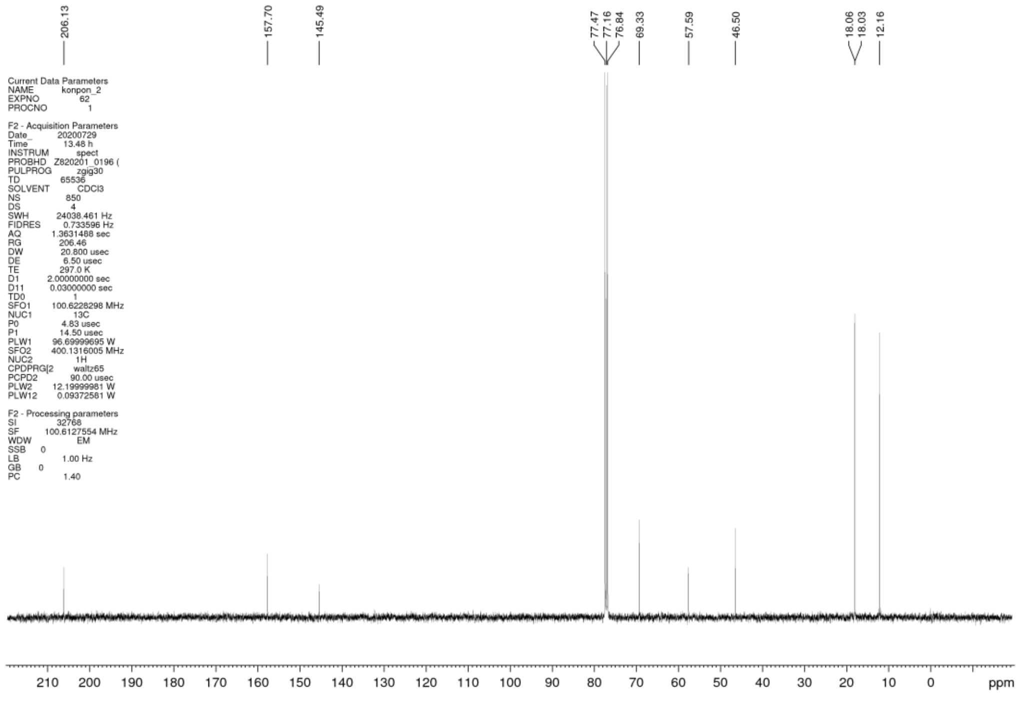
206.13
157.70
145.49
77.47
77.16
76.84
69.33
57.59
46.50
18.06
18.03
12.16
Current Data Parameters
NAME konpon_2
EXPNO 62
PROCNO 1
F2 - Acquisition Parameters
Date_ 20200729
Time 13.48 h
INSTRUM spect
PROBHD Z820201_0196 (
PULPROG zgig30
TD 65536
SOLVENT CDCl3
NS 850
DS 4
SWH 24038.461 Hz
FIDRES 0.733596 Hz
AQ 1.3631488 sec
RG 206.46
DW 20.800 usec
DE 6.50 usec
TE 297.0 K
D1 2.00000000 sec
D11 0.03000000 sec
TD0 1
SFO1 100.6228298 MHz
NUC1 13C
P0 4.83 usec
P1 14.50 usec
PLW1 96.69999695 W
SFO2 400.1316005 MHz
NUC2 1H
CPDPRG[2 waltz65
PCPD2 90.00 usec
PLW2 12.19999981 W
PLW12 0.09372581 W
F2 - Processing parameters
SI 32768
SF 100.6127554 MHz
WDW EM
SSB 0
LB 1.00 Hz
GB 0
PC 1.40
210
200
190
180
170
160
150
140
130
120
110
100
90
80
70
60
50
40
30
20
10
0
ppm

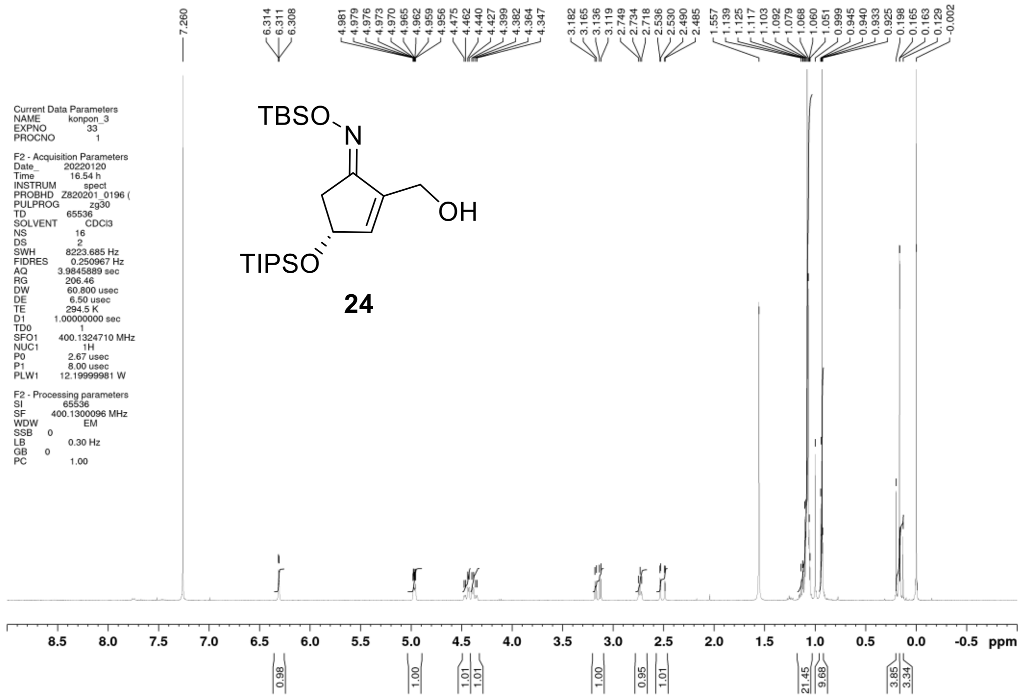
7.260
6.314
6.311
6.308
4.981
4.979
4.976
4.973
4.970
4.965
4.962
4.959
4.956
4.475
4.462
4.440
4.427
4.399
4.382
4.364
4.347
3.182
3.165
3.136
3.119
2.749
2.734
2.718
2.536
2.530
2.490
2.485
1.557
1.139
1.125
1.117
1.103
1.092
1.079
1.068
1.060
1.051
0.999
0.945
0.940
0.933
0.925
0.198
0.165
0.163
0.129
-0.002
Current Data Parameters
NAME konpon_3
EXPNO 33
PROCNO 1
F2 - Acquisition Parameters
Date_ 20220120
Time 16.54 h
INSTRUM spect
PROBHD Z820201_0196 (
PULPROG zg30
TD 65536
SOLVENT CDCl3
NS 16
DS 2
SWH 8223.685 Hz
FIDRES 0.250967 Hz
AQ 3.9845889 sec
RG 206.46
DW 60.800 usec
DE 6.50 usec
TE 294.5 K
D1 1.00000000 sec
TD0 1
SFO1 400.1324710 MHz
NUC1 1H
P0 2.67 usec
P1 8.00 usec
PLW1 12.19999981 W
F2 - Processing parameters
SI 65536
SF 400.1300096 MHz
WDW EM
SSB 0
LB 0.30 Hz
GB 0
PC 1.00
8.5 8.0 7.5 7.0 6.5 6.0 5.5 5.0 4.5 4.0 3.5 3.0 2.5 2.0 1.5 1.0 0.5 0.0 -0.5 ppm
0.98
1.00
1.01
1.01
1.00
0.95
1.01
21.45
9.68
3.85
3.34

TBSO
N
OH
TIPSO

24

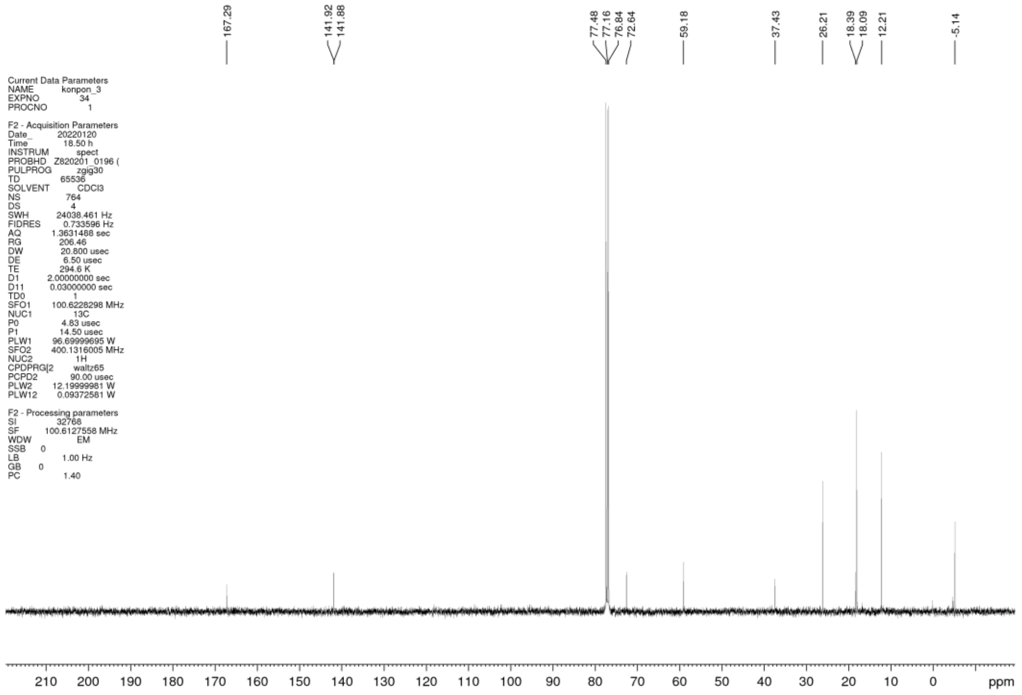
167.29
141.92
141.88
77.48
77.16
76.84
72.64
59.18
37.43
26.21
18.39
18.09
12.21
-5.14
Current Data Parameters
NAME konpon_3
EXPNO 34
PROCNO 1
F2 - Acquisition Parameters
Date_ 20220120
Time 18.50 h
INSTRUM spect
PROBHD Z820201_0196 (
PULPROG zgig30
TD 65536
SOLVENT CDCl3
NS 764
DS 4
SWH 24038.461 Hz
FIDRES 0.733596 Hz
AQ 1.3631488 sec
RG 206.46
DW 20.800 usec
DE 6.50 usec
TE 294.6 K
D1 2.00000000 sec
D11 0.03000000 sec
TD0 1
SFO1 100.6228298 MHz
NUC1 13C
P0 4.83 usec
P1 14.50 usec
PLW1 96.69999695 W
SFO2 400.1316005 MHz
NUC2 1H
CPDPRG[2 waltz65
PCPD2 90.00 usec
PLW2 12.19999981 W
PLW12 0.09372581 W
F2 - Processing parameters
SI 32768
SF 100.6127558 MHz
WDW EM
SSB 0
LB 1.00 Hz
GB 0
PC 1.40
210 200 190 180 170 160 150 140 130 120 110 100 90 80 70 60 50 40 30 20 10 0 ppm

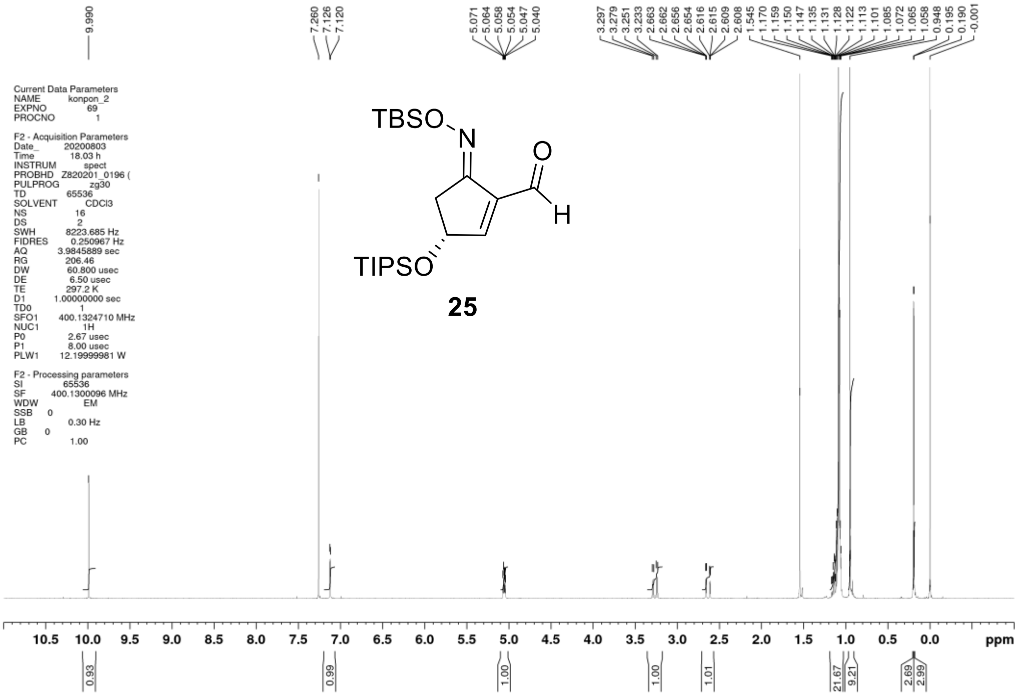
9.990
7.260
7.126
7.120
5.071
5.064
5.058
5.054
5.047
5.040
3.297
3.279
3.251
3.233
2.663
2.662
2.656
2.654
2.616
2.615
2.609
2.608
1.545
1.170
1.159
1.150
1.147
1.135
1.131
1.128
1.122
1.113
1.101
1.085
1.072
1.065
1.058
0.948
0.195
0.190
-0.001
Current Data Parameters
NAME konpon_2
EXPNO 69
PROCNO 1
F2 - Acquisition Parameters
Date_ 20200803
Time 18.03 h
INSTRUM spect
PROBHD Z820201_0196 (
PULPROG zg30
TD 65536
SOLVENT CDCl3
NS 16
DS 2
SWH 8223.685 Hz
FIDRES 0.250967 Hz
AQ 3.9845889 sec
RG 206.46
DW 60.800 usec
DE 6.50 usec
TE 297.2 K
D1 1.00000000 sec
TD0 1
SFO1 400.1324710 MHz
NUC1 1H
P0 2.67 usec
P1 8.00 usec
PLW1 12.19999981 W
F2 - Processing parameters
SI 65536
SF 400.1300096 MHz
WDW EM
SSB 0
LB 0.30 Hz
GB 0
PC 1.00
TBSO
N
O
H
TIPSO
25
10.5
10.0
9.5
9.0
8.5
8.0
7.5
7.0
6.5
6.0
5.5
5.0
4.5
4.0
3.5
3.0
2.5
2.0
1.5
1.0
0.5
0.0
ppm
0.93
0.99
1.00
1.00
1.01
21.67
9.21
2.69
2.99

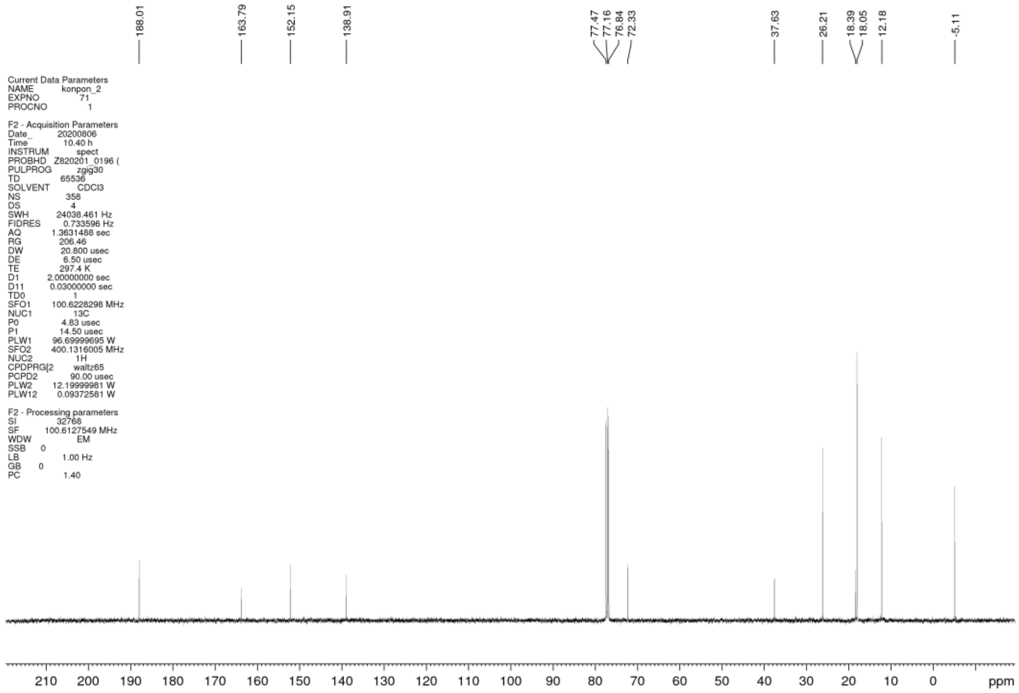
188.01
163.79
152.15
138.91
77.47
77.16
76.84
72.33
37.63
26.21
18.39
18.05
12.18
-5.11
Current Data Parameters
NAME konpon_2
EXPNO 71
PROCNO 1
F2 - Acquisition Parameters
Date_ 20200806
Time 10.40 h
INSTRUM spect
PROBHD Z820201_0196 (
PULPROG zgig30
TD 65536
SOLVENT CDCl3
NS 358
DS 4
SWH 24038.461 Hz
FIDRES 0.733596 Hz
AQ 1.3631488 sec
RG 206.46
DW 20.800 usec
DE 6.50 usec
TE 297.4 K
D1 2.00000000 sec
D11 0.03000000 sec
TD0 1
SFO1 100.6228298 MHz
NUC1 13C
P0 4.83 usec
P1 14.50 usec
PLW1 96.69999695 W
SFO2 400.1316005 MHz
NUC2 1H
CPDPRG[2 waltz65
PCPD2 90.00 usec
PLW2 12.19999981 W
PLW12 0.09372581 W
F2 - Processing parameters
SI 32768
SF 100.6127549 MHz
WDW EM
SSB 0
LB 1.00 Hz
GB 0
PC 1.40
210
200
190
180
170
160
150
140
130
120
110
100
90
80
70
60
50
40
30
20
10
0
ppm

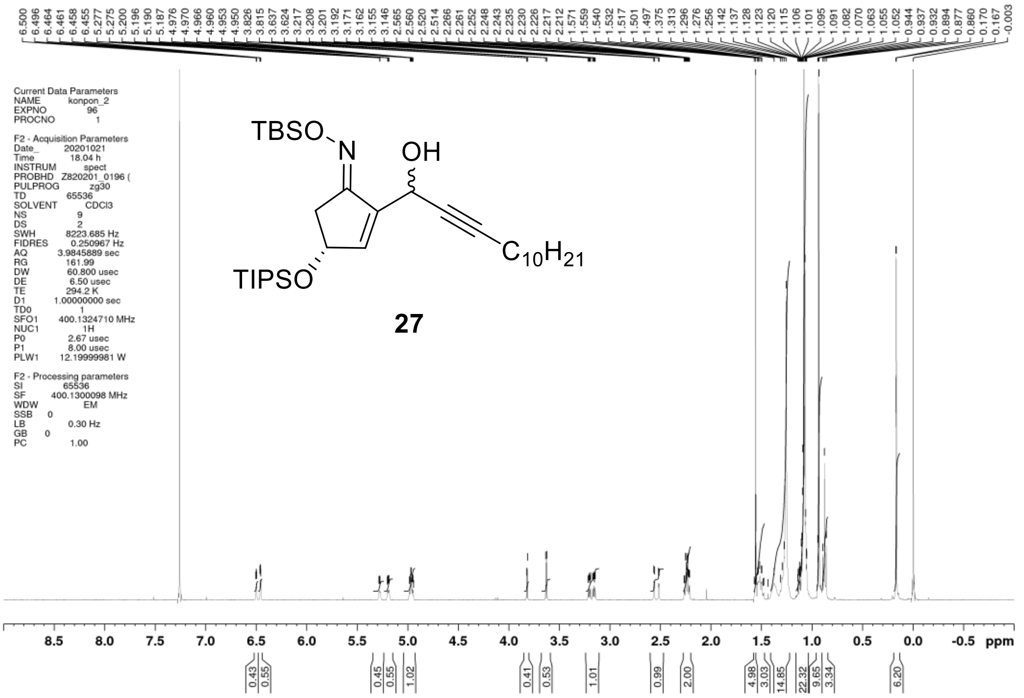
6.500 6.496 6.464 6.461 6.458 6.455 5.277 5.275 5.200 5.196 5.190 5.187 4.976 4.970 4.966 4.960 4.953 4.950 3.826 3.815 3.637 3.624 3.217 3.208 3.201 3.192 3.171 3.162 3.155 3.146 2.565 2.560 2.520 2.514 2.266 2.261 2.252 2.248 2.243 2.235 2.230 2.226 2.217 2.212 1.571 1.559 1.540 1.532 1.517 1.501 1.497 1.375 1.313 1.296 1.276 1.256 1.142 1.137 1.128 1.123 1.120 1.115 1.106 1.101 1.095 1.091 1.082 1.070 1.063 1.055 1.052 0.944 0.937 0.932 0.894 0.877 0.860 0.170 0.167 -0.003
Current Data Parameters
NAME konpon_2
EXPNO 96
PROCNO 1
F2 - Acquisition Parameters
Date_ 20201021
Time 18.04 h
INSTRUM spect
PROBHD Z820201_0196 (
PULPROG zg30
TD 65536
SOLVENT CDCl3
NS 9
DS 2
SWH 8223.685 Hz
FIDRES 0.250967 Hz
AQ 3.9845889 sec
RG 161.99
DW 60.800 usec
DE 6.50 usec
TE 294.2 K
D1 1.00000000 sec
TD0 1
SFO1 400.1324710 MHz
NUC1 1H
P0 2.67 usec
P1 8.00 usec
PLW1 12.19999981 W
F2 - Processing parameters
SI 65536
SF 400.1300098 MHz
WDW EM
SSB 0
LB 0.30 Hz
GB 0
PC 1.00
TBSO
N
OH
TIPSO
C10H21
27
8.5 8.0 7.5 7.0 6.5 6.0 5.5 5.0 4.5 4.0 3.5 3.0 2.5 2.0 1.5 1.0 0.5 0.0 -0.5 ppm
0.43 0.55 0.45 0.55 1.02 0.41 0.53 1.01 0.99 2.00 4.98 3.03 14.85 22.32 9.65 3.34 6.20

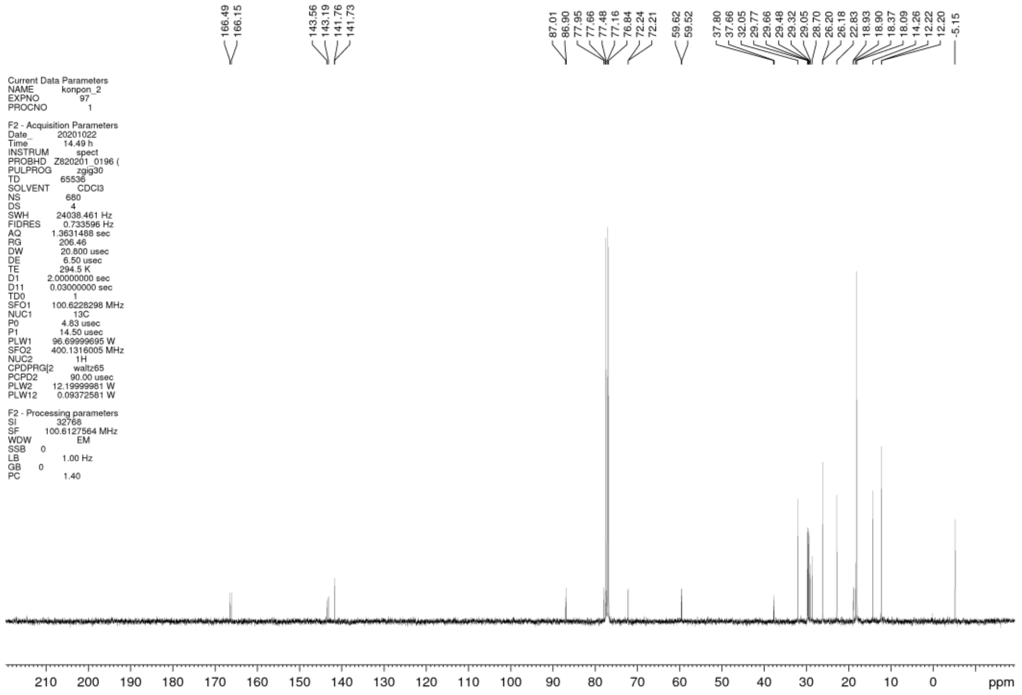
166.49 166.15 143.56 143.19 141.76 141.73 87.01 86.90 77.95 77.66 77.48 77.16 76.84 72.24 72.21 59.62 59.52 37.80 37.66 32.05 29.77 29.66 29.48 29.32 29.05 28.70 26.20 26.18 22.83 18.93 18.90 18.37 18.09 14.26 12.22 12.20 -5.15
Current Data Parameters
NAME konpon_2
EXPNO 97
PROCNO 1
F2 - Acquisition Parameters
Date_ 20201022
Time 14.49 h
INSTRUM spect
PROBHD Z820201_0196 (
PULPROG zgig30
TD 65536
SOLVENT CDCl3
NS 680
DS 4
SWH 24038.461 Hz
FIDRES 0.733596 Hz
AQ 1.3631488 sec
RG 206.46
DW 20.800 usec
DE 6.50 usec
TE 294.5 K
D1 2.00000000 sec
D11 0.03000000 sec
TD0 1
SFO1 100.6228298 MHz
NUC1 13C
P0 4.83 usec
P1 14.50 usec
PLW1 96.69999695 W
SFO2 400.1316005 MHz
NUC2 1H
CPDPRG[2 waltz65
PCPD2 90.00 usec
PLW2 12.19999981 W
PLW12 0.09372581 W
F2 - Processing parameters
SI 32768
SF 100.6127564 MHz
WDW EM
SSB 0
LB 1.00 Hz
GB 0
PC 1.40
210 200 190 180 170 160 150 140 130 120 110 100 90 80 70 60 50 40 30 20 10 0 ppm

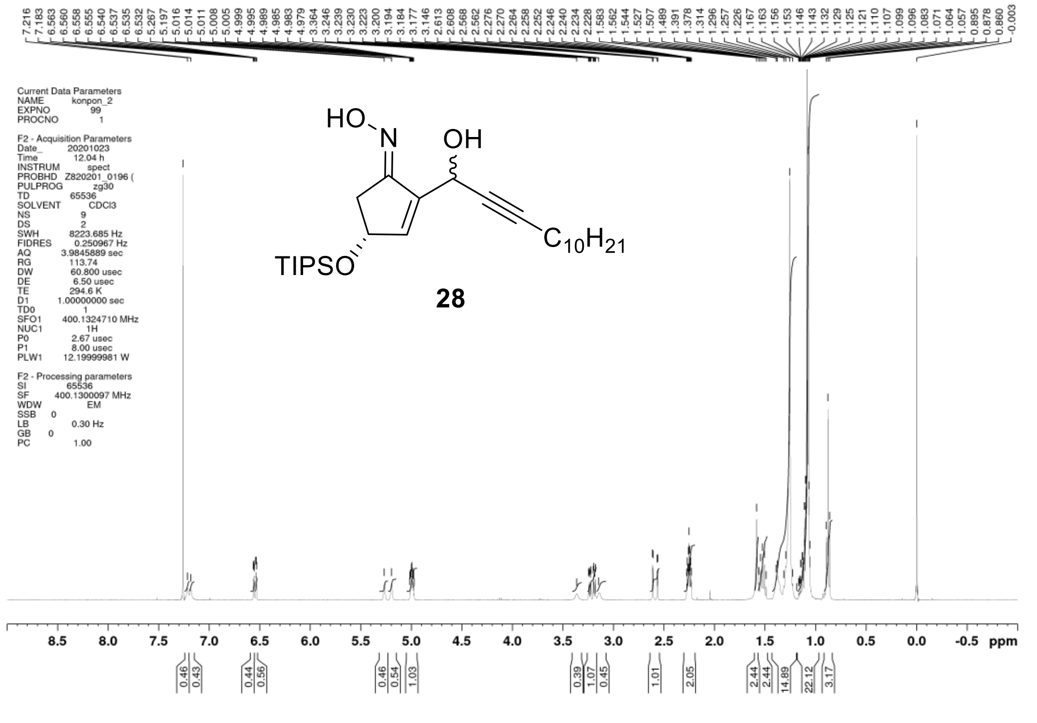
HO
N
OH
TIPSO
C10H21
28

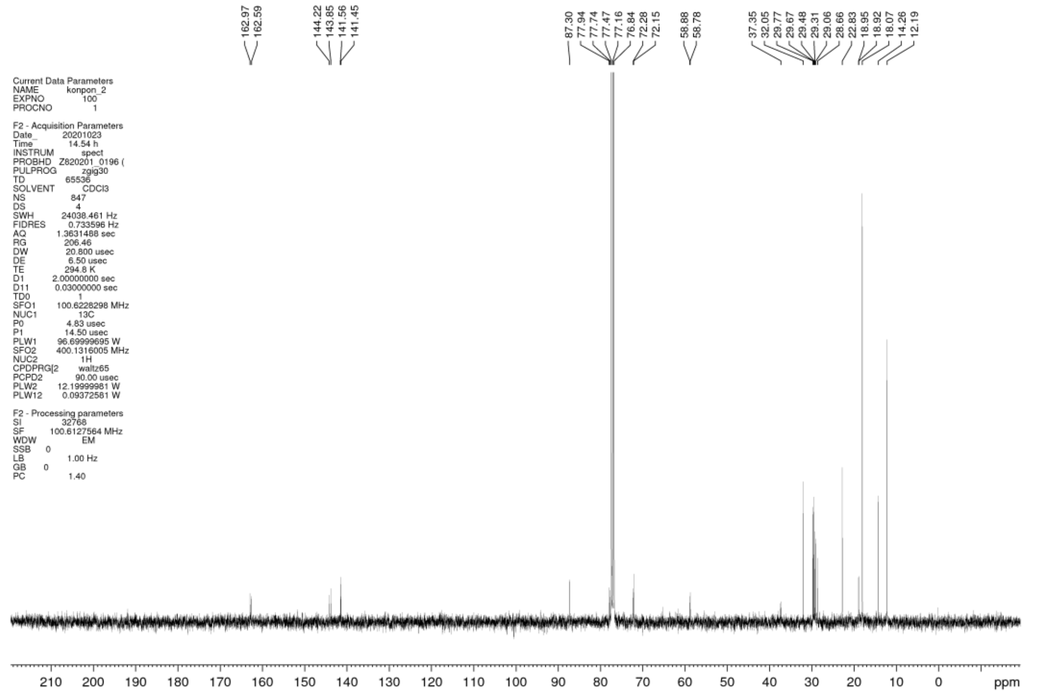

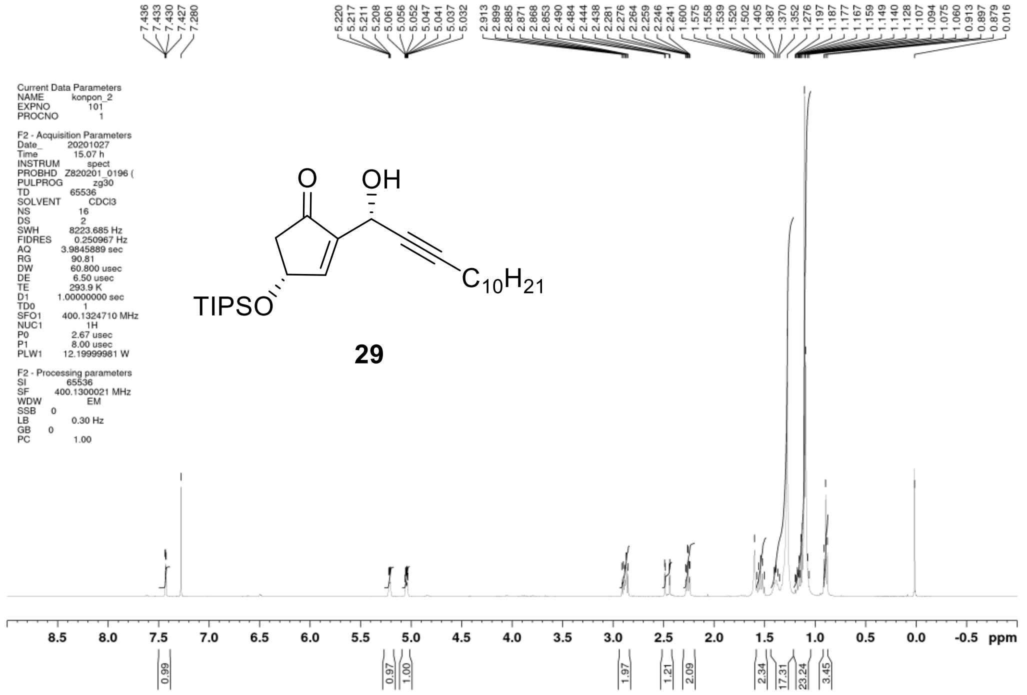

7.436
7.433
7.430
7.427
7.280
5.220
5.217
5.211
5.208
5.061
5.056
5.052
5.047
5.041
5.037
5.032
2.913
2.899
2.885
2.871
2.868
2.853
2.490
2.484
2.444
2.438
2.281
2.276
2.264
2.259
2.246
2.241
1.600
1.575
1.558
1.539
1.520
1.502
1.405
1.387
1.370
1.352
1.276
1.197
1.187
1.177
1.167
1.159
1.149
1.140
1.128
1.107
1.094
1.075
1.060
0.913
0.897
0.879
0.016
Current Data Parameters
NAME konpon_2
EXPNO 101
PROCNO 1
F2 - Acquisition Parameters
Date_ 20201027
Time 15.07 h
INSTRUM spect
PROBHD Z820201_0196 (
PULPROG zg30
TD 65536
SOLVENT CDCl3
NS 16
DS 2
SWH 8223.685 Hz
FIDRES 0.250967 Hz
AQ 3.9845889 sec
RG 90.81
DW 60.800 usec
DE 6.50 usec
TE 293.9 K
D1 1.00000000 sec
TD0 1
SFO1 400.1324710 MHz
NUC1 1H
P0 2.67 usec
P1 8.00 usec
PLW1 12.19999981 W
F2 - Processing parameters
SI 65536
SF 400.1300021 MHz
WDW EM
SSB 0
LB 0.30 Hz
GB 0
PC 1.00
O
OH
TIPSO
$C_{10}H_{21}$
29
8.5 8.0 7.5 7.0 6.5 6.0 5.5 5.0 4.5 4.0 3.5 3.0 2.5 2.0 1.5 1.0 0.5 0.0 -0.5 ppm
0.99
0.97
1.00
1.97
1.21
2.09
2.34
17.31
23.24
3.45

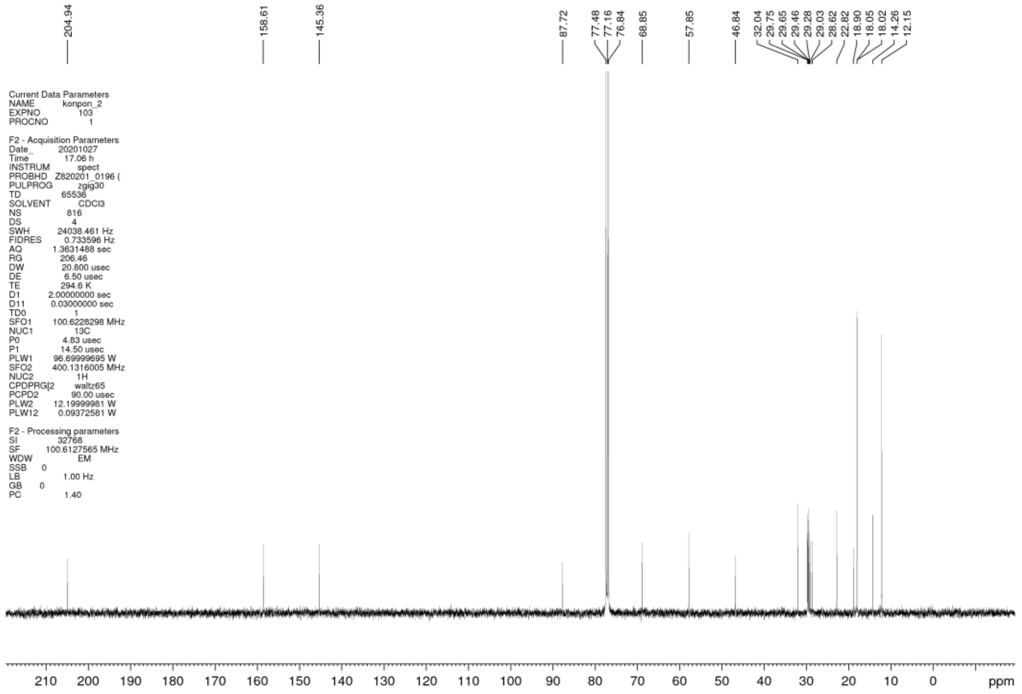

204.94
158.61
145.36
87.72
77.48
77.16
76.84
68.85
57.85
46.84
32.04
29.75
29.65
29.46
29.28
29.03
28.62
22.82
18.90
18.05
18.02
14.26
12.15
Current Data Parameters
NAME konpon_2
EXPNO 103
PROCNO 1
F2 - Acquisition Parameters
Date_ 20201027
Time 17.06 h
INSTRUM spect
PROBHD Z820201_0196 (
PULPROG zgig30
TD 65536
SOLVENT CDCl3
NS 816
DS 4
SWH 24038.461 Hz
FIDRES 0.733596 Hz
AQ 1.3631488 sec
RG 206.46
DW 20.800 usec
DE 6.50 usec
TE 294.6 K
D1 2.00000000 sec
D11 0.03000000 sec
TD0 1
SFO1 100.6228298 MHz
NUC1 13C
P0 4.83 usec
P1 14.50 usec
PLW1 96.69999695 W
SFO2 400.1316005 MHz
NUC2 1H
CPDPRG[2 waltz65
PCPD2 90.00 usec
PLW2 12.19999981 W
PLW12 0.09372581 W
F2 - Processing parameters
SI 32768
SF 100.6127565 MHz
WDW EM
SSB 0
LB 1.00 Hz
GB 0
PC 1.40
210 200 190 180 170 160 150 140 130 120 110 100 90 80 70 60 50 40 30 20 10 0 ppm

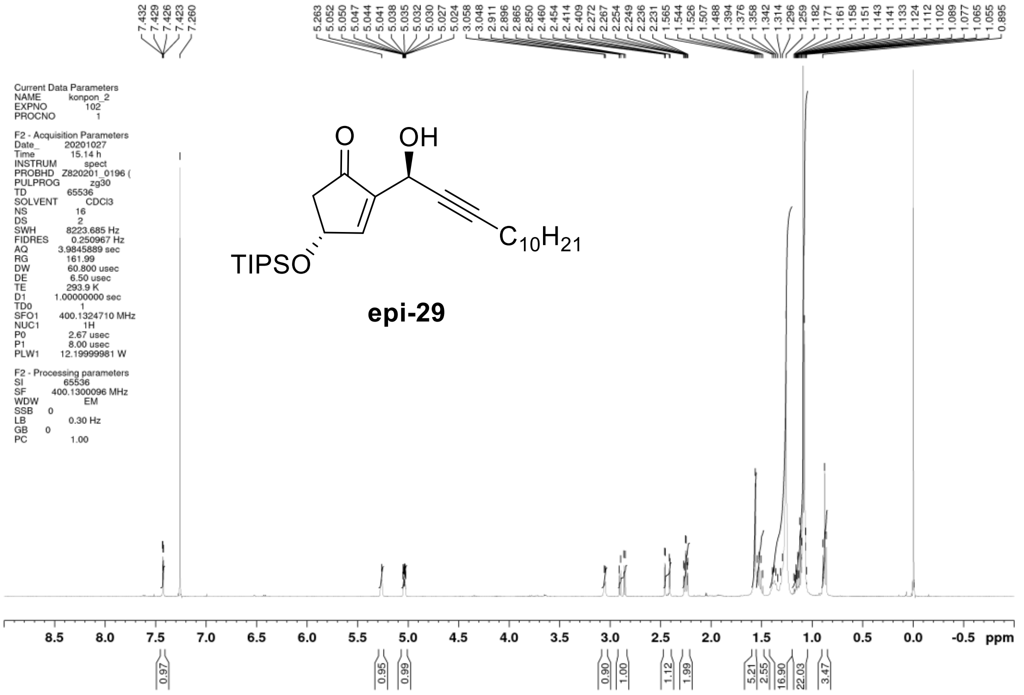
O
OH
TIPSO
C10H21
epi-29
ppm

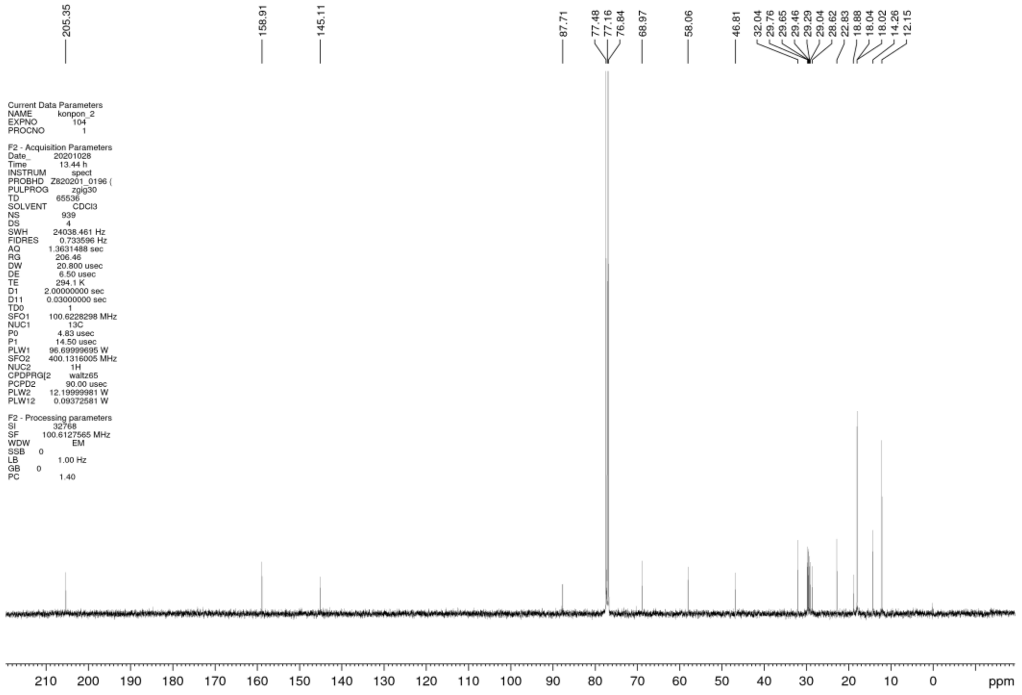
ppm

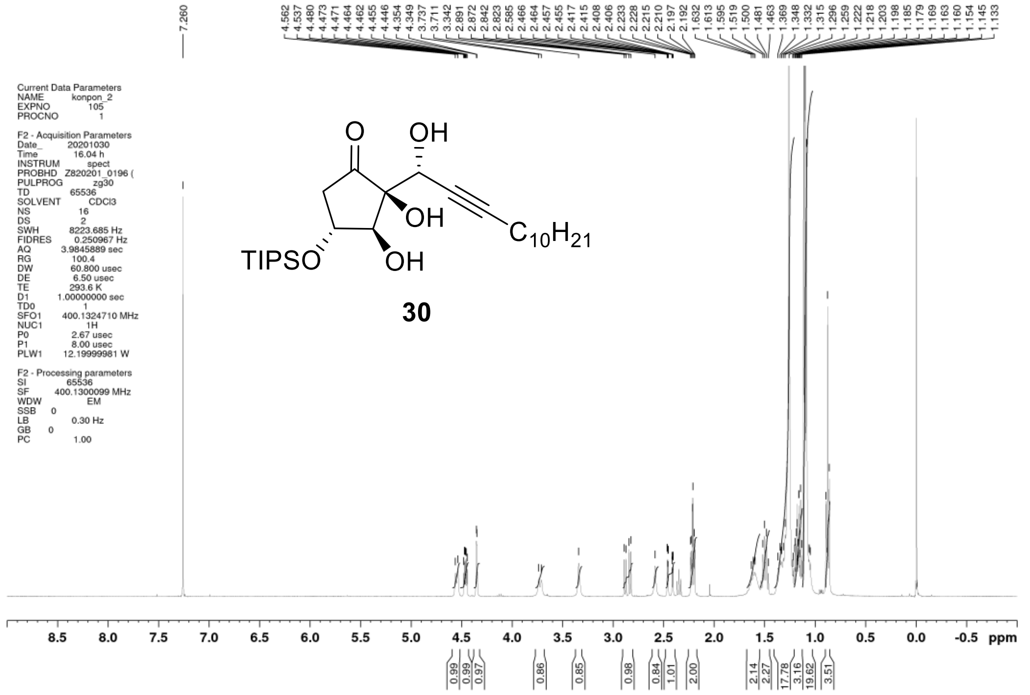
7.260
4.562 4.537 4.480 4.473 4.471 4.464 4.462 4.455 4.446 4.354 4.349 3.737 3.711 3.342 2.891 2.872 2.842 2.823 2.585 2.466 2.464 2.457 2.455 2.417 2.415 2.408 2.406 2.233 2.228 2.215 2.210 2.197 2.192 1.632 1.613 1.595 1.519 1.500 1.481 1.463 1.369 1.348 1.332 1.315 1.296 1.259 1.222 1.218 1.203 1.198 1.185 1.179 1.169 1.163 1.160 1.154 1.145 1.133
Current Data Parameters
NAME konpon_2
EXPNO 105
PROCNO 1
F2 - Acquisition Parameters
Date_ 20201030
Time 16.04 h
INSTRUM spect
PROBHD Z820201_0196 (
PULPROG zg30
TD 65536
SOLVENT CDCl3
NS 16
DS 2
SWH 8223.685 Hz
FIDRES 0.250967 Hz
AQ 3.9845889 sec
RG 100.4
DW 60.800 usec
DE 6.50 usec
TE 293.6 K
D1 1.00000000 sec
TD0 1
SFO1 400.1324710 MHz
NUC1 1H
P0 2.67 usec
P1 8.00 usec
PLW1 12.19999981 W
F2 - Processing parameters
SI 65536
SF 400.1300099 MHz
WDW EM
SSB 0
LB 0.30 Hz
GB 0
PC 1.00
O
OH
OH
TIPSO
OH
$C_{10}H_{21}$
30
8.5 8.0 7.5 7.0 6.5 6.0 5.5 5.0 4.5 4.0 3.5 3.0 2.5 2.0 1.5 1.0 0.5 0.0 -0.5 ppm
0.99 0.99 0.97 0.86 0.85 0.98 0.84 1.01 2.00 2.14 2.27 17.78 3.16 19.62 3.51

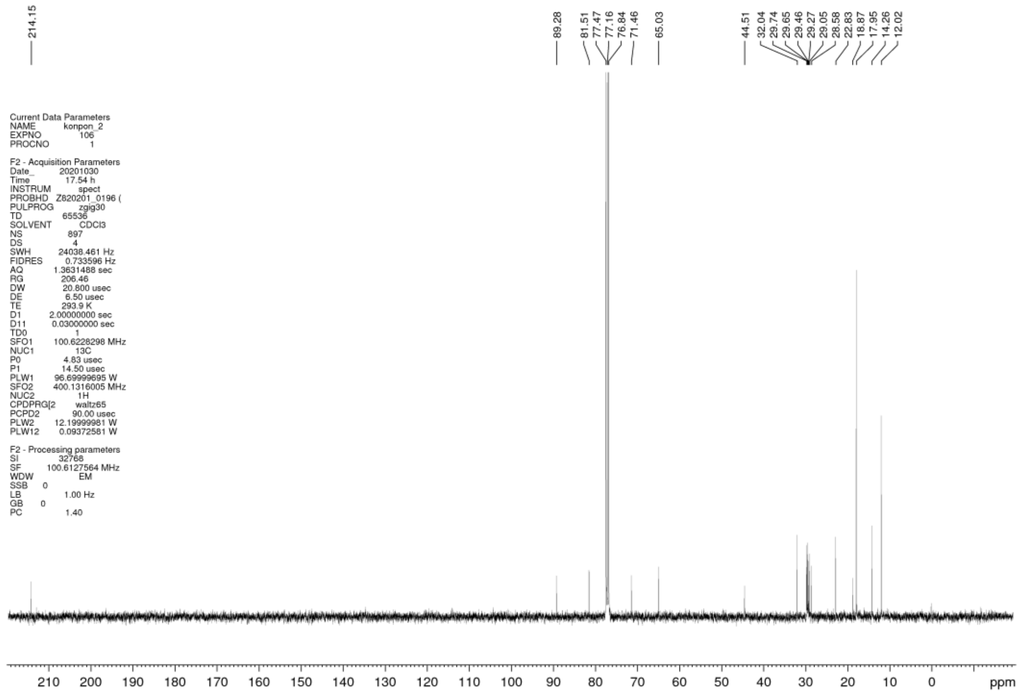
214.15
89.28 81.51 77.47 77.16 76.84 71.46 65.03
44.51 32.04 29.74 29.65 29.46 29.27 29.05 28.58 22.83 18.87 17.95 14.26 12.02
Current Data Parameters
NAME konpon_2
EXPNO 106
PROCNO 1
F2 - Acquisition Parameters
Date_ 20201030
Time 17.54 h
INSTRUM spect
PROBHD Z820201_0196 (
PULPROG zgig30
TD 65536
SOLVENT CDCl3
NS 897
DS 4
SWH 24038.461 Hz
FIDRES 0.733596 Hz
AQ 1.3631488 sec
RG 206.46
DW 20.800 usec
DE 6.50 usec
TE 293.9 K
D1 2.00000000 sec
D11 0.03000000 sec
TD0 1
SFO1 100.6228298 MHz
NUC1 13C
P0 4.83 usec
P1 14.50 usec
PLW1 96.69999695 W
SFO2 400.1316005 MHz
NUC2 1H
CPDPRG[2 waltz65
PCPD2 90.00 usec
PLW2 12.19999981 W
PLW12 0.09372581 W
F2 - Processing parameters
SI 32768
SF 100.6127564 MHz
WDW EM
SSB 0
LB 1.00 Hz
GB 0
PC 1.40
210 200 190 180 170 160 150 140 130 120 110 100 90 80 70 60 50 40 30 20 10 0 ppm

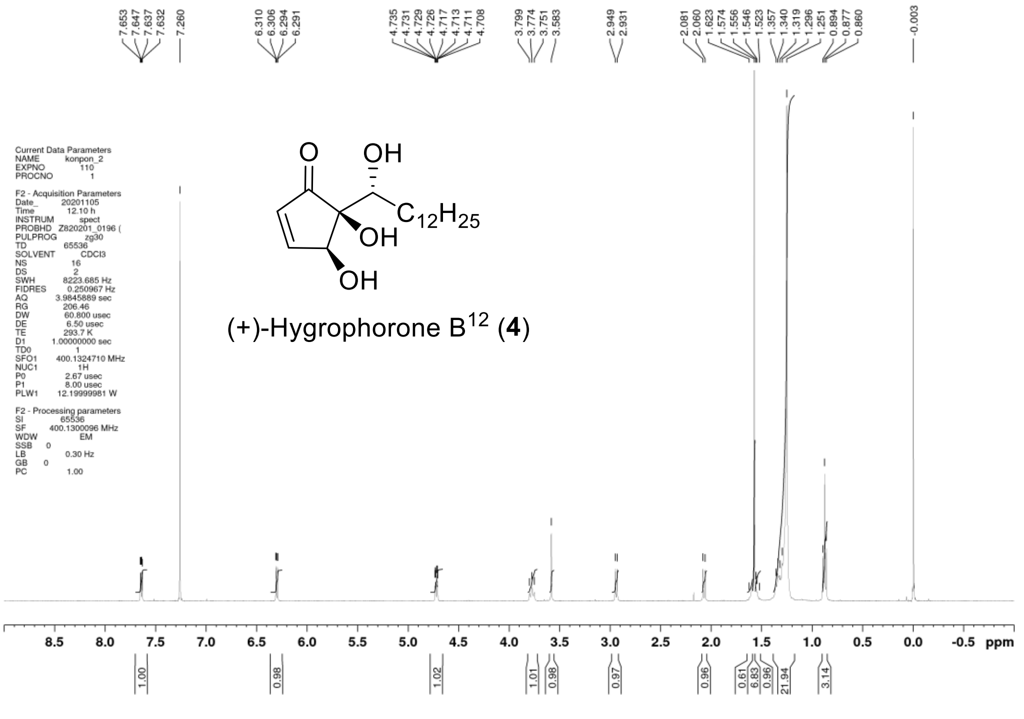

(+)-Hygrophorone B[12] (**4**)

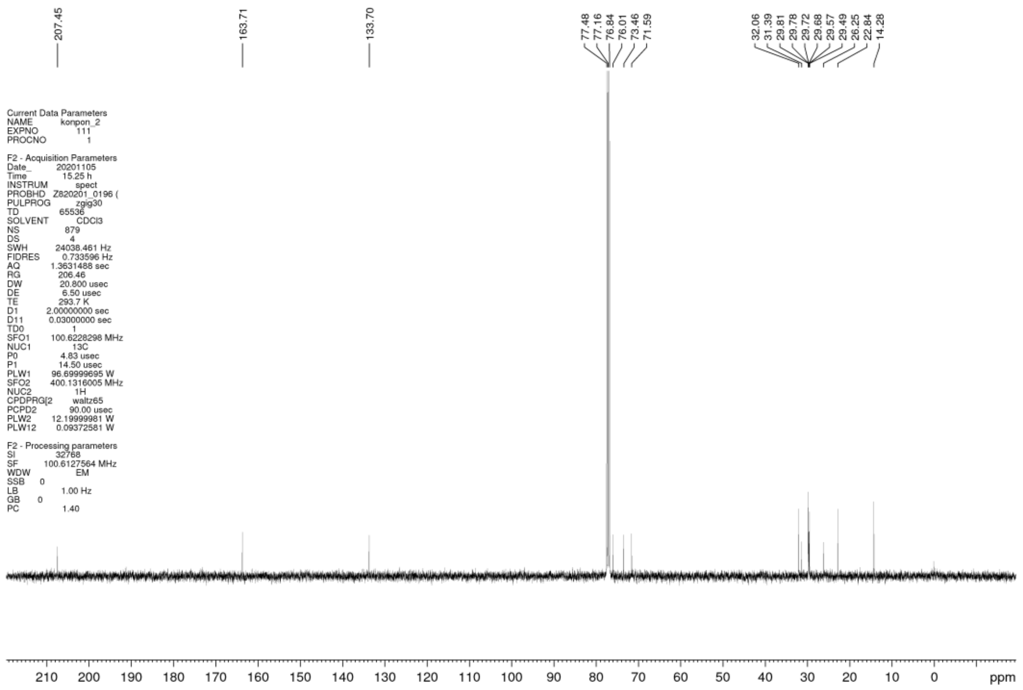

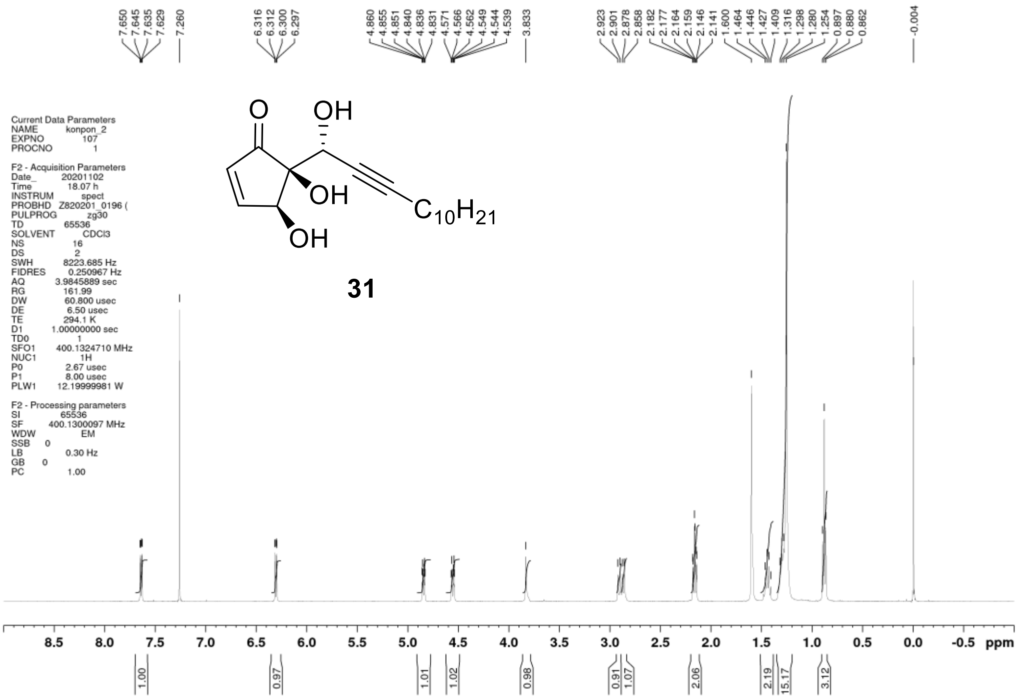

7.650
7.645
7.635
7.629
7.260
6.316
6.312
6.300
6.297
4.860
4.855
4.851
4.840
4.836
4.831
4.571
4.566
4.562
4.549
4.544
4.539
3.833
2.923
2.901
2.878
2.858
2.182
2.177
2.164
2.159
2.146
2.141
1.600
1.464
1.446
1.427
1.409
1.316
1.298
1.280
1.254
0.897
0.880
0.862
-0.004
Current Data Parameters
NAME konpon_2
EXPNO 107
PROCNO 1
F2 - Acquisition Parameters
Date_ 20201102
Time 18.07 h
INSTRUM spect
PROBHD Z820201_0196 (
PULPROG zg30
TD 65536
SOLVENT CDCl3
NS 16
DS 2
SWH 8223.685 Hz
FIDRES 0.250967 Hz
AQ 3.9845889 sec
RG 161.99
DW 60.800 usec
DE 6.50 usec
TE 294.1 K
D1 1.00000000 sec
TD0 1
SFO1 400.1324710 MHz
NUC1 1H
P0 2.67 usec
P1 8.00 usec
PLW1 12.19999981 W
F2 - Processing parameters
SI 65536
SF 400.1300097 MHz
WDW EM
SSB 0
LB 0.30 Hz
GB 0
PC 1.00
O
OH
OH
OH
$C_{10}H_{21}$
31
8.5 8.0 7.5 7.0 6.5 6.0 5.5 5.0 4.5 4.0 3.5 3.0 2.5 2.0 1.5 1.0 0.5 0.0 -0.5 ppm
1.00
0.97
1.01
1.02
0.98
0.91
1.07
2.06
2.19
15.17
3.12

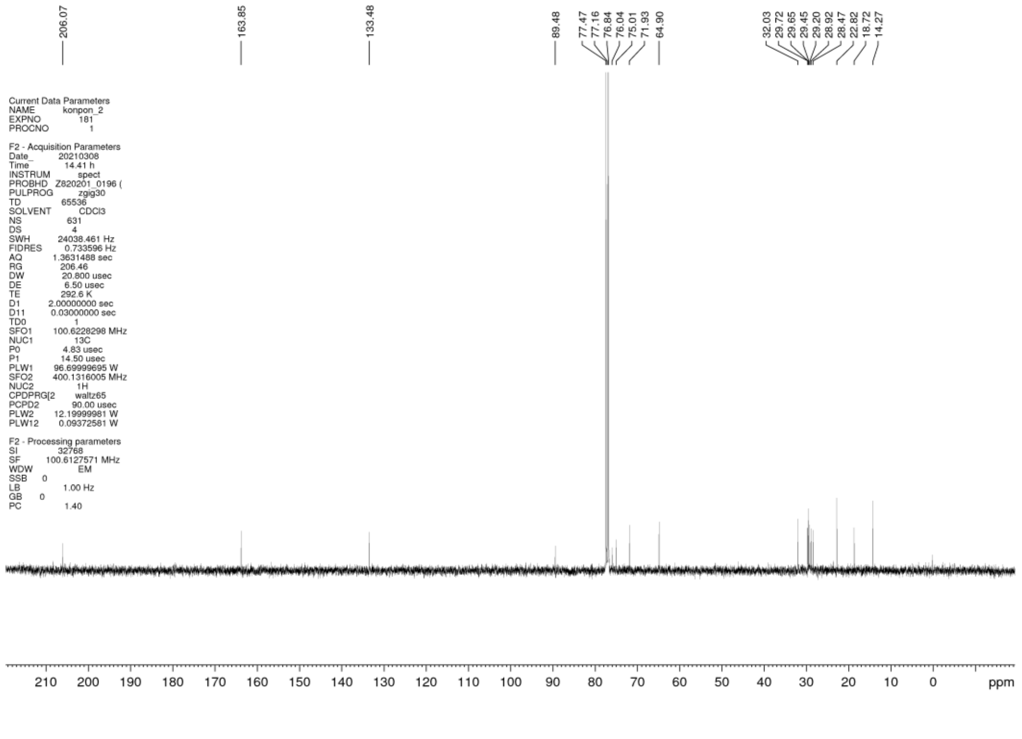

206.07
163.85
133.48
89.48
77.47
77.16
76.84
76.04
75.01
71.93
64.90
32.03
29.72
29.65
29.45
29.20
28.92
28.47
22.82
18.72
14.27
Current Data Parameters
NAME konpon_2
EXPNO 181
PROCNO 1
F2 - Acquisition Parameters
Date_ 20210308
Time 14.41 h
INSTRUM spect
PROBHD Z820201_0196 (
PULPROG zgig30
TD 65536
SOLVENT CDCl3
NS 631
DS 4
SWH 24038.461 Hz
FIDRES 0.733596 Hz
AQ 1.3631488 sec
RG 206.46
DW 20.800 usec
DE 6.50 usec
TE 292.6 K
D1 2.00000000 sec
D11 0.03000000 sec
TD0 1
SFO1 100.6228298 MHz
NUC1 13C
P0 4.83 usec
P1 14.50 usec
PLW1 96.69999695 W
SFO2 400.1316005 MHz
NUC2 1H
CPDPRG[2 waltz65
PCPD2 90.00 usec
PLW2 12.19999981 W
PLW12 0.09372581 W
F2 - Processing parameters
SI 32768
SF 100.6127571 MHz
WDW EM
SSB 0
LB 1.00 Hz
GB 0
PC 1.40
210 200 190 180 170 160 150 140 130 120 110 100 90 80 70 60 50 40 30 20 10 0 ppm

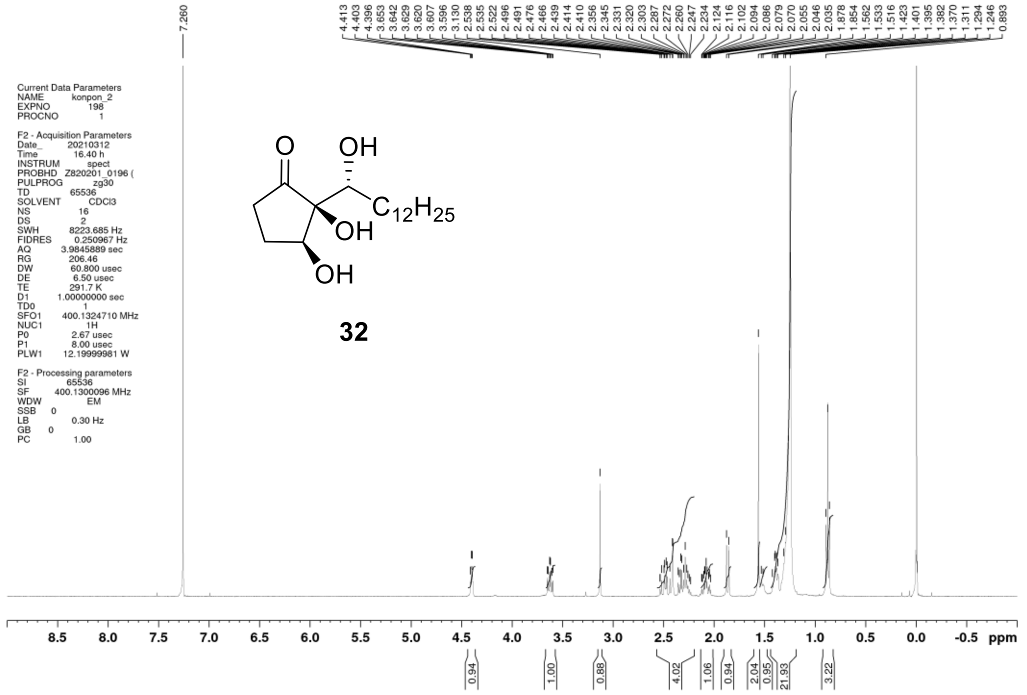
7.260
4.413 4.403 4.396 3.653 3.642 3.629 3.620 3.607 3.596 3.130 2.538 2.535 2.522 2.496 2.491 2.476 2.466 2.439 2.414 2.410 2.356 2.345 2.331 2.320 2.303 2.287 2.272 2.260 2.247 2.234 2.124 2.116 2.102 2.094 2.086 2.079 2.070 2.055 2.046 2.035 1.878 1.854 1.562 1.533 1.516 1.423 1.401 1.395 1.382 1.370 1.311 1.294 1.246 0.893
Current Data Parameters
NAME konpon_2
EXPNO 198
PROCNO 1
F2 - Acquisition Parameters
Date_ 20210312
Time 16.40 h
INSTRUM spect
PROBHD Z820201_0196 (
PULPROG zg30
TD 65536
SOLVENT CDCl3
NS 16
DS 2
SWH 8223.685 Hz
FIDRES 0.250967 Hz
AQ 3.9845889 sec
RG 206.46
DW 60.800 usec
DE 6.50 usec
TE 291.7 K
D1 1.00000000 sec
TD0 1
SFO1 400.1324710 MHz
NUC1 1H
P0 2.67 usec
P1 8.00 usec
PLW1 12.19999981 W
F2 - Processing parameters
SI 65536
SF 400.1300096 MHz
WDW EM
SSB 0
LB 0.30 Hz
GB 0
PC 1.00
O
OH
C12H25
OH
OH
32
8.5 8.0 7.5 7.0 6.5 6.0 5.5 5.0 4.5 4.0 3.5 3.0 2.5 2.0 1.5 1.0 0.5 0.0 -0.5 ppm
0.94 1.00 0.88 4.02 1.06 0.94 2.04 0.95 21.93 3.22

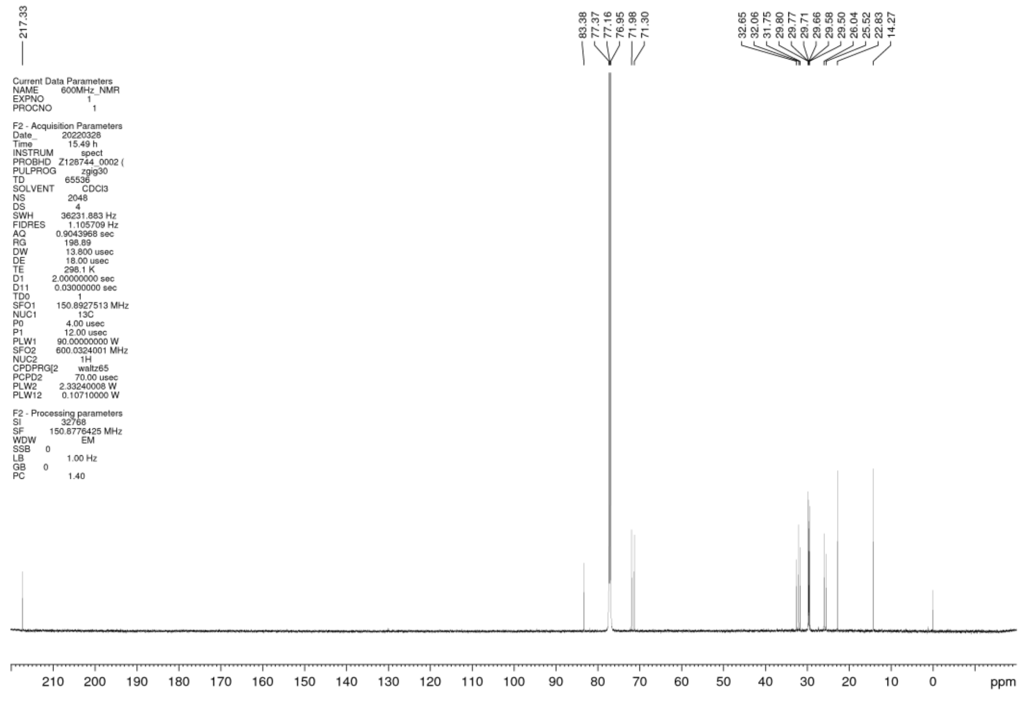
217.33
83.38 77.37 77.16 76.95 71.98 71.30
32.65 32.06 31.75 29.80 29.77 29.71 29.66 29.58 29.50 26.04 25.52 22.83 14.27
Current Data Parameters
NAME 600MHz_NMR
EXPNO 1
PROCNO 1
F2 - Acquisition Parameters
Date_ 20220328
Time 15.49 h
INSTRUM spect
PROBHD Z128744_0002 (
PULPROG zgig30
TD 65536
SOLVENT CDCl3
NS 2048
DS 4
SWH 36231.883 Hz
FIDRES 1.105709 Hz
AQ 0.9043968 sec
RG 198.89
DW 13.800 usec
DE 18.00 usec
TE 298.1 K
D1 2.00000000 sec
D11 0.03000000 sec
TD0 1
SFO1 150.8927513 MHz
NUC1 13C
P0 4.00 usec
P1 12.00 usec
PLW1 90.00000000 W
SFO2 600.0324001 MHz
NUC2 1H
CPDPRG[2 waltz65
PCPD2 70.00 usec
PLW2 2.33240008 W
PLW12 0.10710000 W
F2 - Processing parameters
SI 32768
SF 150.8776425 MHz
WDW EM
SSB 0
LB 1.00 Hz
GB 0
PC 1.40
210 200 190 180 170 160 150 140 130 120 110 100 90 80 70 60 50 40 30 20 10 0 ppm
